# Endogenous Nmnat2 Is an Essential Survival Factor for Maintenance of Healthy Axons

**DOI:** 10.1371/journal.pbio.1000300

**Published:** 2010-01-26

**Authors:** Jonathan Gilley, Michael P. Coleman

**Affiliations:** The Babraham Institute, Babraham Research Campus, Cambridge, United Kingdom; Stanford University School of Medicine

## Abstract

The molecular triggers for axon degeneration remain unknown. We identify endogenous Nmnat2 as a labile axon survival factor whose constant replenishment by anterograde axonal transport is a limiting factor for axon survival. Specific depletion of Nmnat2 is sufficient to induce Wallerian-like degeneration of uninjured axons which endogenous Nmnat1 and Nmnat3 cannot prevent. Nmnat2 is by far the most labile Nmnat isoform and is depleted in distal stumps of injured neurites before Wallerian degeneration begins. Nmnat2 turnover is equally rapid in injured *Wld* ^S^ neurites, despite delayed neurite degeneration, showing it is not a consequence of degeneration and also that Wld^S^ does not stabilize Nmnat2. Depletion of Nmnat2 below a threshold level is necessary for axon degeneration since exogenous Nmnat2 can protect injured neurites when expressed at high enough levels to overcome its short half-life. Furthermore, proteasome inhibition slows both Nmnat2 turnover and neurite degeneration. We conclude that endogenous Nmnat2 prevents spontaneous degeneration of healthy axons and propose that, when present, the more long-lived, functionally related Wld^S^ protein substitutes for Nmnat2 loss after axon injury. Endogenous Nmnat2 represents an exciting new therapeutic target for axonal disorders.

## Abstract 2

We conclude that endogenous Nmnat2 prevents spontaneous degeneration of healthy axons and propose that, when present, the more long-lived, functionally related WldS protein substitutes for Nmnat2 loss after axon injury. Endogenous Nmnat2 represents an exciting new therapeutic target for axonal disorders.

## Author Summary

In a normally functioning neuron, the cell body supplies the axon with materials needed to keep it healthy. This complex logistical activity breaks down completely after injury and often becomes compromised in neurodegenerative diseases, leading to degeneration of the isolated axon. Whilst there are probably many important cargoes delivered from the cell body that isolated axons cannot exist without indefinitely, proteins that are short-lived will be depleted first, so loss of these proteins is likely to act as a trigger for degeneration. Using clues from a mutant mouse whose axons are protected from such degeneration, we have identified delivery of Nmnat2, a protein with an important enzyme activity, as a limiting factor in axon survival. Importantly, Nmnat2 is very labile and its levels decline rapidly in injured axons before they start to degenerate. Even uninjured axons degenerate in a similar way without it. These properties are consistent with loss of Nmnat2 being a natural stimulus for axon degeneration, and it might therefore be a suitable target for therapeutic intervention.

## Introduction

The endogenous molecular trigger for Wallerian degeneration remains unknown. Recent progress towards understanding how the slow Wallerian degeneration fusion protein (Wld^S^) delays degeneration of injured and sick axons has not addressed this wider question [1]–[8], and this aberrant protein is only expressed in a few strains of mouse, rat, and fly. Knowledge of the normal regulation of axon survival in wild-type animals should not only lead to greater mechanistic insight but could also have important therapeutic implications for axon protection since pharmacological manipulation of endogenous processes is likely to be more achievable than overexpression of exogenous proteins.

Many stresses that induce Wallerian or Wallerian-like degeneration involve a partial or complete block of axonal transport. Since transport is bi-directional, degeneration could be triggered by failed anterograde delivery of essential survival factors or by failed removal of harmful substances by retrograde transport. Defective anterograde transport seems more directly associated with axon loss than dysfunctional retrograde transport [9]–[13]. Therefore, extending an old model [14], we propose a “survival factor delivery hypothesis” of axon degeneration. We suggest that axon integrity requires continuous anterograde delivery of one or more labile, cell body–synthesized survival factors. Other axonal components should be dispensable, more long-lived, or synthesized locally. Once supply is disrupted, following injury or other insult, levels of the limiting survival factor(s) will drop below a critical threshold due to natural turnover, activating an intrinsic axon degeneration program.

This model has several attractions. First, the initial latent phase of Wallerian degeneration [14],[15] would reflect the rate of survival factor turnover before the critical threshold is reached. Second, altered turnover would explain how low temperature and proteasome inhibition extend this latent phase [16]–[18]. Finally, redistribution of the remaining survival factor(s) in the distal stump by axonal transport could underlie the progressive nature of Wallerian degeneration [14],[19],[20].

No such endogenous survival factor has been identified, but the *Wld* ^S^ protective mechanism offers important clues. Wld^S^ contains the N-terminal 70 amino acids of multiubiquitination factor Ube4b fused, in frame, to NAD^+^ synthesizing enzyme Nmnat1 [21]. Both regions are required for full Wld^S^ function in vivo [4],[5]. The N-terminal VCP binding region probably targets the essential Nmnat activity to a specific subcellular location [2],[5]. Despite being predominantly nuclear [6], recent studies indicate a cytoplasmic and potentially axonal site of action for Wld^S^ [2],[3],[7],[8], rekindling interest in its relationship to the earlier model of a putative endogenous survival factor(s) [14]. Wld^S^, an aberrant protein, cannot be one of these factors, but Nmnat1 [22] and the other mammalian Nmnat isoforms (Nmnat2 and Nmnat3 [23],[24]) are candidates since they all possess the same critical enzyme activity and can all delay axon degeneration in primary neuronal culture when expressed exogenously at high levels [1],[25],[26]. Only Nmnat3 has so far been shown to confer robust protection to injured axons in vivo when wild-type proteins (except for a tag used for detection) are overexpressed [2],[4],[8].

In support of the “survival factor delivery hypothesis” we show that briefly suppressing protein synthesis in cell bodies of uninjured primary neuronal cultures induces Wallerian-like degeneration. The ability of a single protein (Wld^S^) to block this suggests that only one or a few critical factors are directly involved. We hypothesized that Wld^S^ substitutes for one or more mammalian Nmnat isoforms, so we compared their properties against those predicted for a critical axon survival factor. We reasoned that depletion should trigger Wallerian-like degeneration without injury, its natural half-life should be consistent with the latent phase of Wallerian degeneration (Wld^S^ should be much more stable to extend this period), the survival factor should be degraded by the proteasome to explain why proteasome inhibition extends axonal survival, it should be present in axons, and it should significantly prolong injured axon survival when highly overexpressed (to outweigh its short half-life). Nmnat2 uniquely fits this profile, indicating that its depletion after injury is a trigger for Wallerian degeneration and that “dying-back” pathology is likely to reflect defects in Nmnat2 axonal transport or synthesis.

## Results

### Somatic Protein Synthesis Suppression Induces Wallerian-Like Degeneration

**Figure 1:**
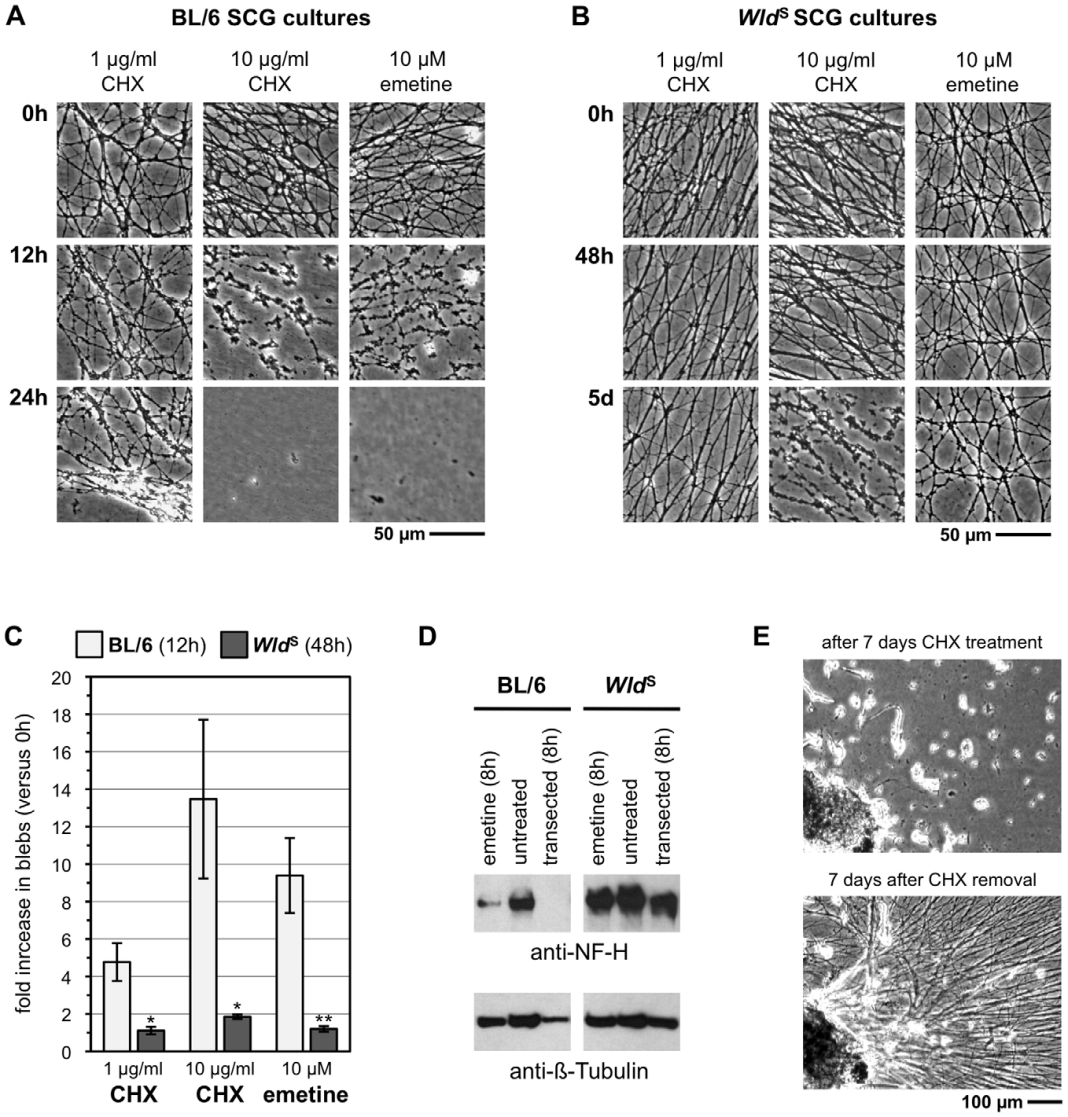
Protein synthesis suppression induces rapid Wallerian-like degeneration of SCG neurites before loss of neuronal viability. Representative bright-field images of distal neurites from wild-type (BL/6) (A) and *Wld* ^S^ (B) mouse SCG explant cultures treated with 1 or 10 µg/ml CHX or 10 µM emetine as indicated. Images of the same field of distal neurites were captured at the times indicated on the left. Neurites in DMSO-treated wild-type cultures continue to grow and appear morphologically normal (unpublished data). (C) The fold increase in neurite blebs 12 h after treatment with CHX or emetine in BL/6 cultures and 48 h after treatment in *Wld* ^S^ cultures, relative to the same neurites at the start of the treatment (0 h), was quantified from three or more independent experiments combining data from multiple fields (error bars = ±S.E.M.). Blebbing is significantly reduced in *Wld* ^S^ cultures even at this much later time point (*p<0.05, **p = 0.01, *t* test *Wld* ^S^ at 48 h versus BL/6 at 12 h). (D) Anti-NF-H and anti-ß-Tubulin immunoblots of neurite-only extracts from wild-type or *Wld* ^S^ SCG explant cultures either treated with 10 µM emetine for 8 h, left untreated, or 8 h after cut. Each lane represents neurites collected from SCG cultures containing three ganglia. (E) Bright-field images of a mouse SCG explant after 7 d of treatment with 1 µg/ml CHX and 7 d after CHX removal. Images are representative of three independent experiments.

Our main hypothesis predicts that blocking synthesis of one or more putative axon survival factors should trigger Wallerian-like degeneration without injury, similar to that induced by blocking axonal transport [27],[28]. To test this we initially inhibited all protein translation in mouse superior cervical ganglia (SCG) explant cultures, using two unrelated inhibitors, cycloheximide (CHX) and emetine, to rule out nonspecific effects. One µg/ml CHX, which suppresses global protein synthesis by more than 95% [29],[30], not only stopped neurite outgrowth as expected [30],[31] but also induced widespread blebbing of distal neurites (Figure 1A and 1C). Ten µg/ml CHX or 10 µM emetine caused more rapid and extensive blebbing of neurites, presumably due to more complete suppression of protein synthesis, followed by fragmentation and detachment shortly afterwards (Figure 1A and 1C), similar to the degeneration of transected neurites. To test whether the degeneration is Wallerian-like, we used cultures from slow Wallerian degeneration (*Wld* ^S^) mice and found a delay of over 48 h (Figure 1B and 1C). Similar results with rat SCG cultures and mouse dorsal root ganglion (DRG) cultures indicate that these events are not restricted to one species or neuron type (Figure S1). Delayed degeneration in *Wld* ^S^ cultures after inhibition of translation also shows that local translation of mRNAs in neurites is unlikely to underlie Wld^S^-mediated axon protection as hypothesized previously [32]. Similarly, localized translation is not required in injured neurites for Wld^S^-mediated protection, and it is also not needed for Wallerian degeneration itself (Figure S2).

Rapid cleavage of neurofilament heavy chain (NF-H) is an early molecular change that occurs as injury-induced Wallerian degeneration is initiated after the latent phase both in vitro and in vivo [6],[18]. We found that this also occurs after protein synthesis suppression in wild-type cultures but not in *Wld* ^S^ cultures (Figure 1D). Thus, molecular assays also indicate this degeneration is Wallerian-like.

Importantly, degeneration induced by protein synthesis suppression is not due to loss of neuronal viability but is a much earlier event independent of cell death. Even 7 d after treatment with 1 µg/ml CHX, long after complete degeneration of neurites, many SCG cell bodies retain the ability to re-grow neurites when this reversible inhibitor is removed (Figure 1E). Most cell bodies in 7-d CHX-treated dissociated cultures also excluded Trypan Blue, further indicating neuron viability (unpublished data).

**Figure 2:**
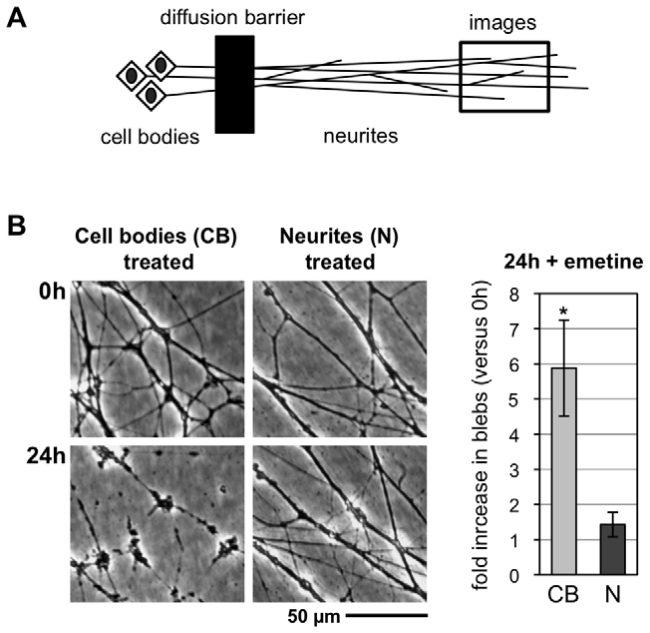
Neurites degenerate when suppression of protein synthesis is restricted to the cell body. (A) Diagram showing the organization of compartmented wild-type mouse SCG cultures. (B) Representative bright-field images of distal neurites from the side chamber of a compartmented culture in which 10 µM emetine was added to either the central chamber (cell bodies treated) or side chamber (neurites treated). Images of the same field of neurites were captured just after emetine addition (0 h) and 24 h later. The fold increase in blebbing at 24 h relative to 0 h (of the same neurites) was quantified from three independent experiments combining data from multiple fields (error bars = ±S.E.M.) and is shown on the right. Treatment of cell bodies (CB) alone induces significantly more blebbing of distal neurites than treatment of the distal neurites (N) themselves (*p = 0.026, *t* test). Comparable results were obtained with 1 µg/ml CHX (unpublished data).

To test directly whether a critical axon survival factor(s) has to be synthesized and delivered from cell bodies, we used compartmented cultures where distal neurites can be treated separately from cell bodies and proximal neurites (Figure 2). Neurites degenerated only when inhibitors were applied to the compartment containing neuronal cell bodies and proximal neurites. Consistent with a previous report [33], translation inhibitors applied only to distal neurites caused no significant degeneration within this timeframe. Indeed, neurites continued to grow (unpublished data). Thus, suppression of protein synthesis in the cell body triggers Wallerian-like neurite degeneration, providing strong support for the survival factor delivery hypothesis and suggesting the survival factor(s) is proteinaceous.

### Nmnat2 Knock-Down Induces Wallerian-Like Degeneration

We then investigated the molecular basis of these findings. Because Nmnat1 contributes essential Nmnat enzyme activity to the Wld^S^ fusion protein, we reasoned that Wld^S^ might protect axons by substituting for injury-induced loss of an endogenous Nmnat activity. Transcripts of all three mammalian Nmnat isoforms are expressed in mouse SCG neurons (Figure S3 and [26]), suggesting each is a reasonable candidate. Moreover, although their predominant localizations are nuclear (Nmnat1), Golgi-associated (Nmnat2) and mitochondrial (Nmnat3) [34], the recent finding that Wld^S^ acts at a non-nuclear site despite its nuclear abundance [3] reminds us that low levels of protein can act elsewhere, especially if enzyme activity amplifies the effect. We therefore decided to test whether any of the Nmnat isoforms possess the predicted properties of an endogenous axon survival factor in our model.

**Figure 3:**
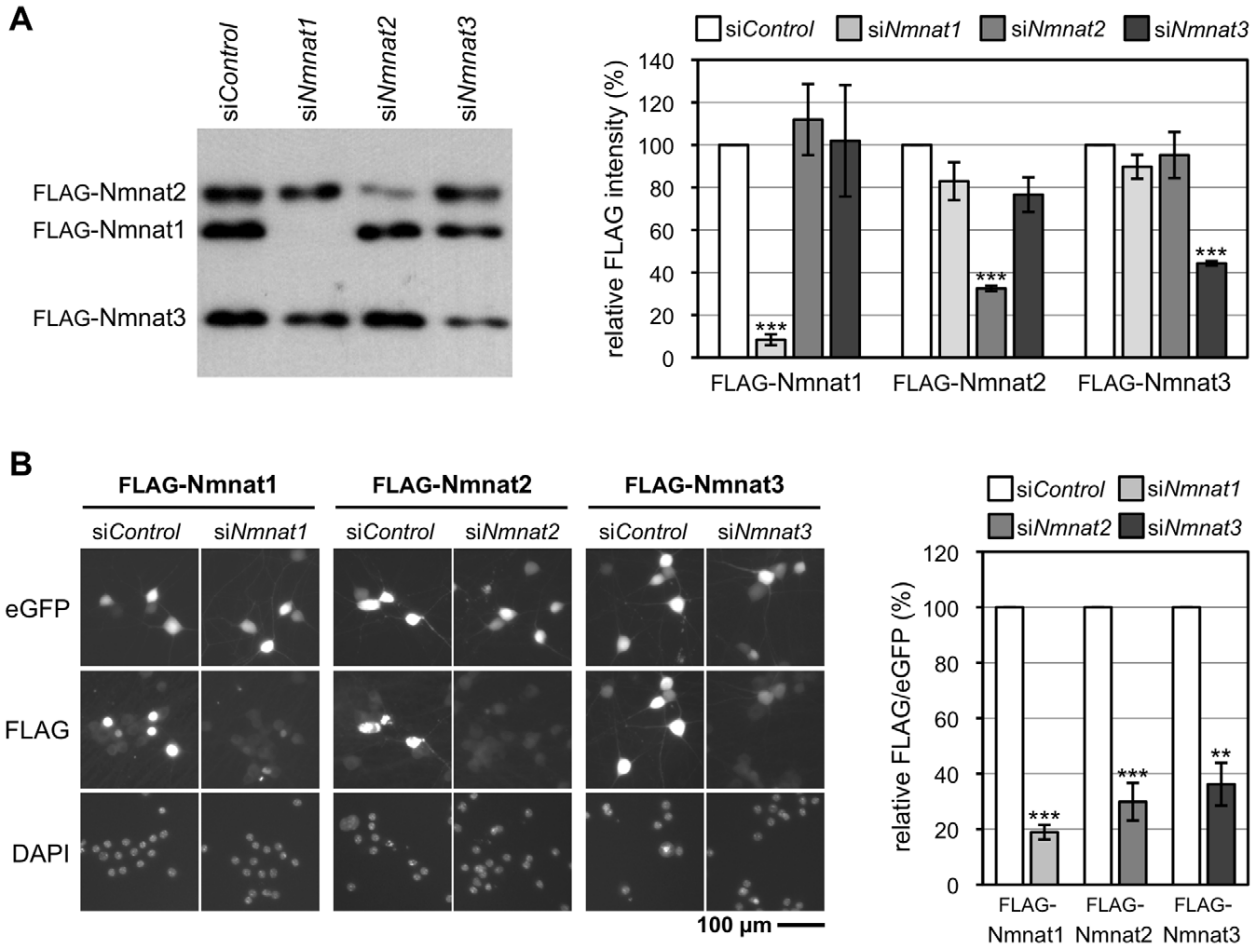
The *Nmnat* siRNA pools are specific for their intended targets. (A) Each *Nmnat* siRNA pool specifically blocks expression of a FLAG-tagged version of its intended target in transfected HEK 293T cells. Representative FLAG immunoblot of cells 24 h after co-transfection with expression vectors for each FLAG-Nmnat isoform together with a non-targeting pool of siRNA (si*Control*) or one of the si*Nmnat* siRNA pools as indicated. Quantification of band intensities relative to bands in the si*Control* lane in three independent experiments is shown on the right (error bars = ±S.E.M.). Each siRNA pool specifically and significantly reduces expression of its target (***p<0.001, *t* test of respective si*Nmnat* versus si*Control*). Each FLAG-Nmnat acts as internal control of transfection efficiency for the others. Only 3/4 of the pooled *Nmnat2* siRNAs and 2/4 of the pooled *Nmnat3* siRNAs target sequences in the respective expression vectors (compared to 4/4 for the *Nmnat1* siRNA pool). All should target the endogenous mRNAs. (B) Each *Nmnat* siRNA pool specifically blocks expression of a FLAG-tagged version of its intended target in injected SCG neurons. Representative fluorescent images of SCG neurons 48 h after co-injection with one FLAG-Nmnat expression vector, pEGFP-C1, and the relevant si*Nmnat* pool or non-targeting siRNA pool (si*Control*). eGFP fluorescence identifies injected neurons, FLAG immunostaining shows expression levels of each FLAG-Nmnat, and DAPI labels nuclei. Each si*Nmnat* siRNA pool significantly reduces expression of its target isoform compared to si*Control* as indicated on the right by quantification of FLAG immunostaining relative to eGFP fluorescence in individual neurons (**p<0.01, ***p<0.001, *t* test of respective si*Nmnat* versus si*Control*). Data are from three independent experiments in which at total of 36–55 injected neurons were analyzed (error bars = ±S.E.M.) Localization of each tagged isoform is consistent with their expected distribution; Nmnat1 in nuclei and Nmnat2 and Nmnat3 in cytoplasmic compartments.

The first key prediction is that survival factor depletion should induce Wallerian-like neurite degeneration without injury as levels drop below a critical threshold. We used pools of siRNAs (si*Nmnat1*, *2*, or *3*) to knock down expression of the murine Nmnat isoforms and confirmed specificity for the appropriate isoform by assessing their ability to prevent expression of N-terminal FLAG-tagged Nmnat (FLAG-Nmnat) proteins in transfected HEK 293T cells and SCG neurons (Figure 3).

**Figure 4:**
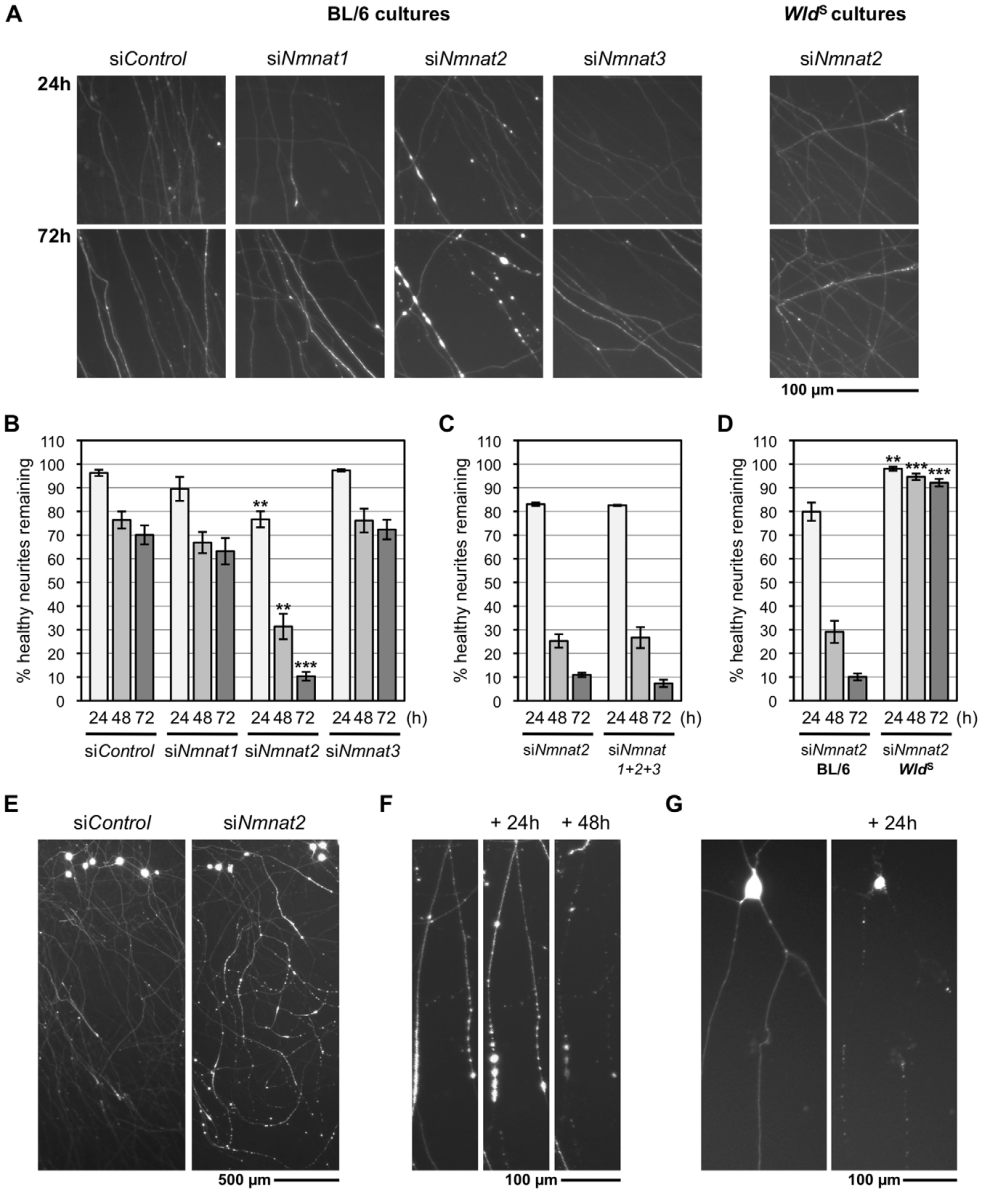
*Nmnat2* siRNA triggers Wallerian-like degeneration of SCG neurites. (A) Representative fluorescent images of the distal ends of (DsRed2-labeled) neurites of wild-type (BL/6) or *Wld* ^S^ SCG neurons 24 and 72 h after injection with non-targeting siRNA (si*Control*) or siRNA targeting each Nmnat isoform (si*Nmnat1*, *2*, *or 3*), as indicated, together with pDsRed2-N1 (50 ng/µl) (see also Figure S4). (B) Relative survival of (DsRed2-labeled) neurites of wild-type SCG neurons injected with si*Control*, si*Nmnat1*, si*Nmnat2*, or si*Nmnat3*. The number of healthy DsRed2-labeled neurites remaining at each time point is shown as a percentage of the total number (both healthy and abnormal) 24 h after injection and was quantified from three independent experiments combining data from multiple fields (error bars = ±S.E.M.). Only si*Nmnat2* causes significant neurite loss (**p<0.01, ***p<0.001, *t* test si*Nmnat2* versus si*Control* at equivalent time points). (C) Co-injection of si*Nmnat1*, *2*, and *3* (each at 100 ng/µl) does not accelerate (wild-type) neurite loss compared to injection of si*Nmnat2* alone (100 ng/µl). Data were quantified as in (B) from three independent experiments (error bars = ±S.E.M.). (D) Neurite loss caused by si*Nmnat2* injection is abolished in *Wld* ^S^ SCG neurons up to 72 h after injection (**p<0.01, ***p<0.001, *t* test *Wld* ^S^ versus BL/6 at equivalent time points). Data were quantified as in (B) from four independent experiments (error bars = ±S.E.M.). (E–G) Representative fluorescent images showing the typical characteristics of the neurite degeneration caused by si*Nmnat2* injection. Multiple DsRed2-positive neuritic swellings are only observed after si*Nmnat2* injection (E) and these precede a characteristic progressive distal-to-proximal “dying-back” neurite degeneration (F). Neurites showing “dying-back” degeneration can be followed back to morphologically normal cell bodies. This contrasts the more rapid, catastrophic neurite degeneration that coincides with cell body death (G) and is seen following injection of all siRNA pools (including si*Control*). Images in (E) were captured 48 h after injection and show injected cell bodies at the top of each panel.

To assess the effect of Nmnat isoform knock-down in SCG neurons, we used a microinjection-based strategy (see Figure S4), enabling us to consistently introduce similar amounts of siRNA. Neurons in wild-type dissociated cultures were first injected with each siRNA pool, with DsRed2 expression allowing visualization of injected neurons and their neurites. Of the three *Nmnat* siRNA pools, only injection of si*Nmnat2* caused a significant reduction in the percentage of healthy neurites compared to the non-targeting siRNA pool (si*Control*) (Figure 4A and 4B). Some of the neurites of the si*Nmnat2*-injected neurons already appeared abnormal 24 h after injection, when the entire lengths of the DsRed2-labeled neurites could first be clearly visualized, and almost all showed abnormal morphology or had completely degenerated 72 h after injection. In contrast, injection of si*Control*, si*Nmnat1*, and si*Nmnat3* all caused relatively little degeneration (Figure 4A and 4B), and neurites continued to grow (unpublished data). Combined injection of all three *Nmnat* siRNA pools did not significantly accelerate neurite degeneration relative to si*Nmnat2* alone (Figure 4C). Thus, Nmnat2 knock-down is sufficient to induce neurite degeneration, whereas knock-down of the other Nmnat isoforms has no clear effect on neurite survival. To confirm that the si*Nmnat2*-induced neurite degeneration is Wallerian-like, we microinjected *Wld* ^S^ neurons with si*Nmnat2* and found degeneration was completely blocked for at least 72 h (Figure 4A and 4D).

To rule out a contribution from any off-target effect of the four individual siRNAs within the si*Nmnat2* pool, we tested whether they could cause neurite degeneration when injected individually or in non-overlapping sub-pools (Figure S5). One siRNA alone (J-059190-11) and two others in combination (J-059190-10 and J-059190-12) triggered significant neurite degeneration that was similar to that induced by the complete pool. A clear combinatorial effect was also seen as J-059190-11 injected at the concentration it contributes to the si*Nmnat2* pool caused significantly less neurite degeneration than the pool itself. Together, these observations show that si*Nmnat2*-induced neurite degeneration is due to knock-down of Nmnat2.

The si*Nmnat2*-induced neurite degeneration is distinctive, characterized by the appearance of multiple neuritic DsRed2-containing swellings and a distal-to-proximal “dying-back” progression that appears to be independent of neuronal viability (Figure 4E and 4F). In contrast, the small amount of background neurite degeneration seen with all the siRNA pools (including si*Control*) coincides with cell death and is faster and morphologically distinct (Figure 4G).

**Figure 5:**
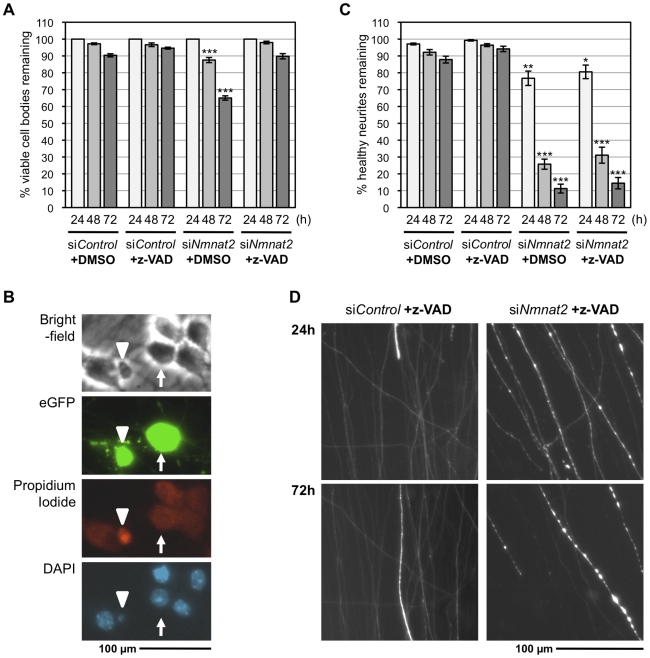
Neurite degeneration following si*Nmnat2* injection precedes loss of neuronal viability. (A) Viability of wild-type SCG neurons injected with non-targeting siRNA (si*Control*) or *Nmnat2* siRNA (si*Nmnat2*), together with pEGFP-C1 (10 ng/µl), and treated with 50 µM z-VAD-fmk or vehicle (DMSO) as indicated. The number of neurons with normal gross morphology remaining at each time point is shown as a percentage of those present at 24 h and was quantified from three independent experiments (error bars = ±S.E.M.). There is a small but significant decrease in the percentage of viable neurons 48 and 72 h after injection with si*Nmnat2* (+DMSO) relative to si*Control* (***p<0.001, *t* test si*Nmnat2* versus si*Control* at equivalent time points), but this is reduced to control levels following treatment with z-VAD-fmk. (B) Assessment of neuronal viability based on gross morphology matches that based on other indicators of viability. At the end of experiments in (A), neurons were counter-stained with propidium iodide (PI), a DNA stain that can only penetrate the membranes of non-viable cells, and DAPI. Abnormal gross morphology seen with bright-field and eGFP imaging correlated precisely with PI staining and nuclear condensation/fragmentation (revealed by DAPI staining) typical of cell death. An arrowhead indicates an abnormal neuron (rarely seen in these experiments) and an arrow a healthy neuron. (C) Quantification of neurite survival corresponding to the analysis of neuronal viability in (A). The number of healthy eGFP-labelled neurites remaining at each time point (quantified from multiple fields in each experiment) is shown as a percentage of the total number (both healthy and abnormal) 24 h after injection (error bars = ±S.E.M.). Significant neurite loss is seen following si*Nmnat2* injection irrespective of treatment with z-VAD-fmk (*p<0.05, **p<0.01, ***p<0.001, *t* test si*Nmnat2* ± z-VAD-fmk versus si*Control* ± z-VAD-fmk at equivalent time points) with no significant difference between the two. (D) Representative fluorescent images of the distal ends of eGFP-labelled neurites of wild-type (BL/6) SCG neurons 24 and 72 h after injection with si*Control* or si*Nmnat2* and treated with z-VAD-fmk.

Some loss of neuronal viability occurred in these experiments, irrespective of the siRNA injected, but a small, additional decrease in neuronal viability following si*Nmnat2* knock-down was also apparent (Figure S6). Even though this reduction in neuronal viability, relative to si*Control*, was proportionately much smaller than the reduction in neurite survival (Figure S6F), we sought to completely exclude the possibility that cell death might be responsible for the si*Nmnat2*-associated neurite degeneration. We were able to almost completely eliminate neuronal cell death in the si*Nmnat2* injection experiments in two ways (Figure 5). First, we reduced expression of the fluorescent marker after finding that toxicity was causing the (caspase-independent) background cell death. Second, we found that the small si*Nmnat2*-associated decrease in neuronal viability could be prevented by the pan-caspase inhibitor z-VAD-fmk (Figure 5A), indicating that this death is caspase-dependent. Importantly, the amount of si*Nmnat2*-induced neurite degeneration was unchanged when cell death was reduced in these ways (compare Figure 5C and 5D to Figures 4A, 4B, and S6). This clearly shows that neurite degeneration precedes any associated loss of neuron viability in these experiments. It is also consistent with *Wld* ^S^-mediated protection of neurites (Figure 4D) being able to reduce si*Nmnat2*-associated neuronal loss to control levels (Figure S6C), despite the fact that Wld^S^ cannot directly prevent neuronal cell death in SCG cultures [35]. In addition, failure of z-VAD-fmk to prevent si*Nmnat2*-induced neurite degeneration provides further evidence that it is Wallerian-like as Wallerian degeneration has been shown to be unaffected by a range of anti-apoptotic interventions [36]–[38].

Thus, constitutive expression of endogenous Nmnat2 in SCG neurons is required to prevent spontaneous “dying-back” Wallerian-like neurite degeneration. Importantly, these data also indicate that endogenous Nmnat1 and Nmnat3 cannot compensate for loss of Nmnat2, despite the ability of these proteins to protect injured neurites when sufficiently overexpressed [1],[25].

### Nmnat2 Is the Most Labile Nmnat Isoform

**Figure 6:**
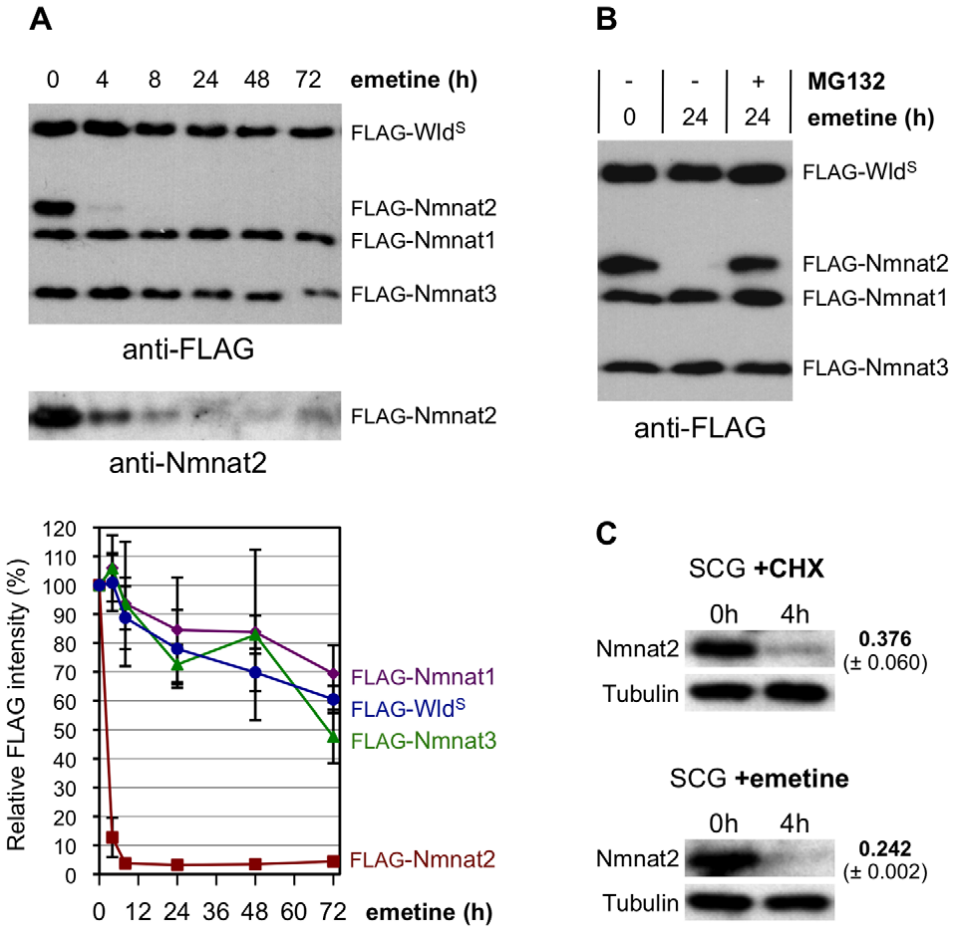
Nmnat2 is the most labile Nmnat isoform and is degraded by the proteasome. (A) Relative stabilities of FLAG-tagged Nmnat isoforms and Wld^S^ in HEK 293T cells co-transfected with expression vectors for each and treated 24 h after transfection with 10 µM emetine to block translation for the times indicated. A representative FLAG immunoblot is shown (top panel). The same blot was re-probed with an Nmnat2 antibody (bottom panel) to show that loss of anti-FLAG signal is primarily due to protein turnover rather than cleavage of the FLAG epitope from the protein. Quantification of band intensities for each protein from three independent experiments is shown below as a percentage of untreated (0 h) band intensities (error bars = ±S.E.M.). Co-transfection allows direct comparison of stabilities of each protein in the same cells. (B) FLAG-Nmnat2 turnover in transfected HEK 293T cells is prevented by proteasome inhibition. Cells were transfected as in (A) and treated with 10 µM emetine for 0 or 24 h, ±20 µM MG-132. A FLAG immunoblot representative of three independent experiments is shown. (C) Endogenous Nmnat2 is rapidly turned over in SCG explant cultures after blocking translation with CHX (1 µg/ml) or emetine (10 µM). Representative immunoblots are shown comparing steady-state levels of Nmnat2 (0 h) with levels after 4 h of protein synthesis suppression. ß-Tubulin acts as a loading control. Nmnat2 band intensity at 4 h is shown as a fraction of that at 0 h after normalization to ß-Tubulin and was quantified from two independent experiments each (error bars = ±S.E.M.). These values are consistent with the different rates at which CHX (1 µg/ml) and emetine induce neurite degeneration (Figure 1).

In our model, axon degeneration is initiated when survival factor levels drop below a critical threshold after synthesis or delivery is blocked. If Nmnat2 depletion acts as a trigger for Wallerian degeneration, Nmnat2 half-life should be compatible with the short latent phase of 4–6 h before transected SCG neurites degenerate. Wld^S^, on the other hand, should be more stable to directly substitute for loss of endogenous Nmnat2. A direct comparison of the relative turnover rates of the FLAG-tagged murine Nmnat isoforms and Wld^S^ in co-transfected HEK 293T cells (Figure 6A) showed that FLAG-tagged Nmnat2 is turned over rapidly when protein synthesis is blocked with an in vitro half-life of less than 4 h. In contrast, there was minimal turnover of FLAG-tagged Wld^S^, Nmnat1, and Nmnat3 up to 72 h. Similar results were also obtained with C-terminal FLAG-tagged proteins (unpublished data). We also found that proteasome inhibition with MG-132 largely prevented turnover of FLAG-tagged Nmnat2 in these cells for at least 24 h (Figure 6B). Importantly, turnover of endogenous Nmnat2 in SCG explants following protein synthesis inhibition was found to be similarly rapid (Figure 6C).

The half-life of Nmnat2 is also consistent with the time when wild-type SCG neurites become committed to degenerate after inhibition of translation (Figure S7A). Neurites exposed to CHX for just 4 h remain healthy and continue to grow for over 5 d, but they become irreversibly committed to degenerate when exposed to CHX for just 8 h, despite only minimal evidence of degeneration when CHX is removed. Intermediate treatment for 6 h gave a mixed outcome. This suggests that degeneration of these neurites can be prevented by reestablishing synthesis of the labile survival factor(s) providing levels have not dropped below a critical threshold. The precise threshold can only be determined when the duration of downstream events leading to activation and execution of degeneration are better understood. Importantly, Wld^S^ expression not only delays the onset of neurite degeneration following protein synthesis suppression, it also delays their commitment to degenerate at least 3-fold (Figure S7B).

Therefore, the half-life of Nmnat2, but not Nmnat1 and Nmnat3, is compatible with its turnover being a trigger for Wallerian degeneration. Furthermore, the longer half-life of Wld^S^ is consistent with it substituting for Nmnat2 loss for a prolonged period.

### Endogenous Nmnat2 Degrades Rapidly and Spontaneously in Injured Neurites

**Figure 7:**
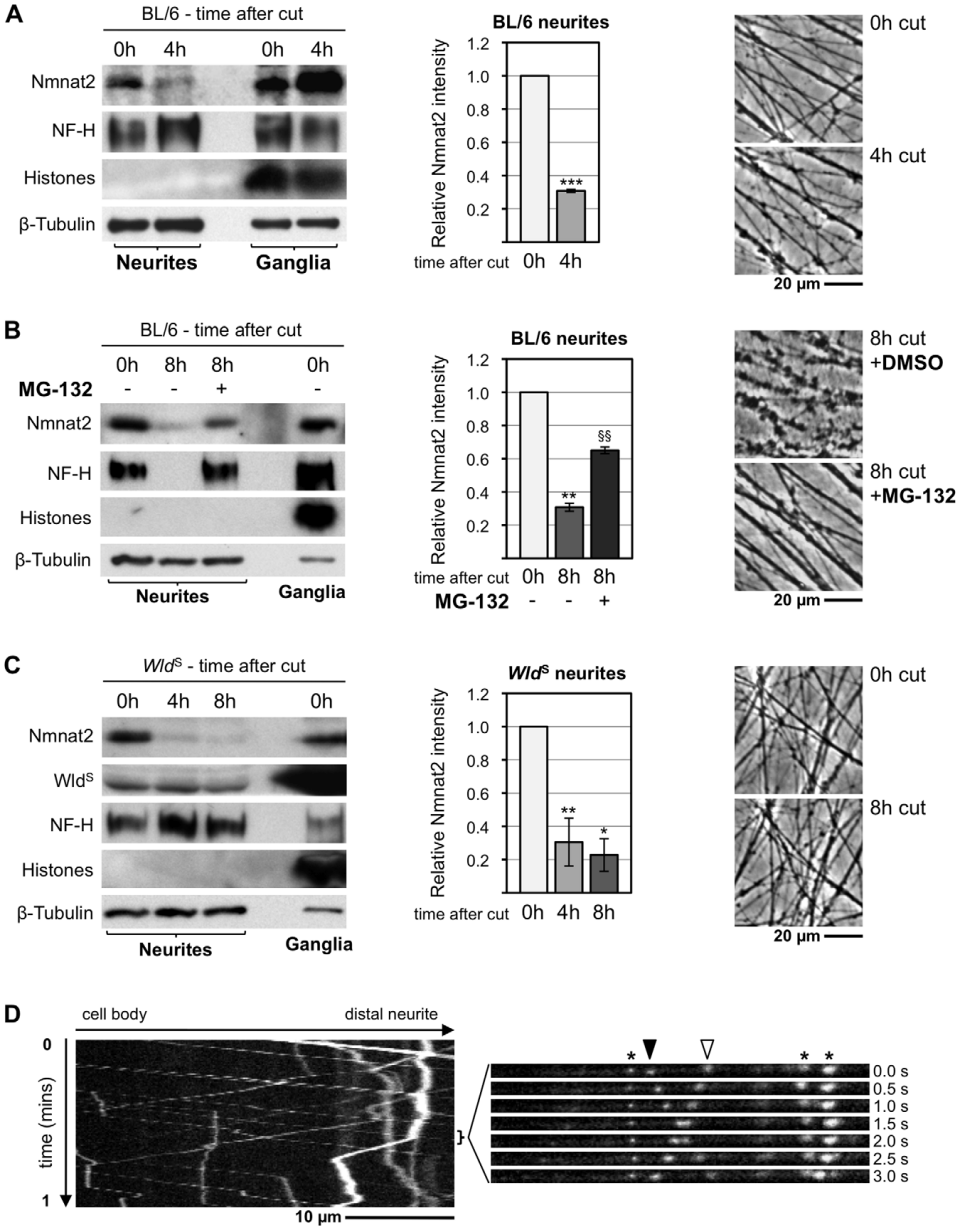
Endogenous Nmnat2 is present in SCG neurites and undergoes rapid turnover after transection. (A–C) Relationship between Nmnat2 turnover in neurites and other parameters of neurite health in wild-type cultures (A), wild-type cultures ±20 µM MG-132 (B), and *Wld* ^S^ cultures (C). Representative immunoblots show detection of Nmnat2, Wld^S^ (where applicable), NF-H, 16 kDa core Histones, and ß-Tubulin just after cut (0 h) and 4 and/or 8 h later. Material collected from SCG explant cultures derived from 15–20 ganglia was needed to detect Nmnat2 in each lane. Loss of the NF-H band is an early consequence of axon degeneration and absence of the 16 kDa core Histones band in neurite extracts confirms there is no detectable contamination with SCG cell bodies or non-neuronal cells. ß-Tubulin represents a loading control. Relative Nmnat2 band intensities in neurite-only lanes are shown as a fraction of levels at 0 h after normalisation to ß-Tubulin and were quantified from two or three independent experiments (error bars = ±S.E.M.). Nmnat2 is significantly depleted in untreated wild-type and *Wld* ^S^ neurites shortly after separation from their ganglia (*p<0.05, **p<0.01, ***p<0.001, *t* test 4 or 8 h versus 0 h). Proteasome inhibition with MG-132 significantly reduces this Nmnat2 loss at 8 h after transection (^§§^ *p*<0.01, *t* test 8 h +MG-132 versus 8 h untreated). Images show representative transected neurite morphology (in the same field where applicable) at the indicated times after cut. (D) Kymograph of the bottom axon in Video S1 showing fast axonal transport of particles containing Nmnat2-eGFP with a bias in the anterograde direction. An image series from the indicated region of the kymograph is shown on the right. It highlights a particle moving anterogradely (filled arrowhead), one moving retrogradely (empty arrowhead), and several stationary particles (asterisks).

According to our model, the putative axon survival factor should also be present in neurites under normal conditions, and its level in transected neurites should drop significantly prior to initiation of degeneration at 4–6 h. Therefore, we assessed Nmnat2 levels in neurite-only extracts from SCG explant cultures at the time of transection and 4 h afterwards when the gross morphology of the transected neurites still appears relatively normal (Figure 7A). Neurite extracts contained significant amounts of Nmnat2 at the time of transection and this fell to ∼30% of steady-state levels within 4 h. Furthermore, loss of endogenous Nmnat2 occurs before cleavage of NF-H, which accompanies physical break-down of SCG neurites after injury [18] or protein synthesis suppression (Figure 1D), and before β-Tubulin degradation. An increase in Nmnat2 levels in the corresponding cell body/proximal neurite extracts 4 h after separation of their transected distal neurites is also seen. This probably represents accumulation of Nmnat2 in a greatly reduced cellular volume (see Discussion).

Proteasome inhibition modestly extends the latent phase of Wallerian degeneration in SCG explant cultures [18], so we tested whether this correlates with reduced turnover of endogenous Nmnat2 given that FLAG-tagged Nmnat2 is degraded via the proteasome in HEK cells (Figure 6B). Neurites treated with the proteasome inhibitor MG-132 appear relatively normal 8 h after transection, with no associated NF-H cleavage, whereas untreated neurites show extensive physical and molecular signs of degeneration (Figure 7B). We found that loss of Nmnat2 was also significantly reduced by MG-132 at this time (Figure 7B), consistent with depletion of endogenous Nmnat2 being a critical trigger for axon degeneration. The fact that Nmnat2 turnover was not completely prevented might explain why the duration of neurite protection by MG-132 is fairly limited [18], although prolonged proteasome inhibition is also toxic to axons [39].

Nmnat2 loss within 4 h in transected wild-type neurites seems unlikely to be a consequence of axon degeneration, as cytoskeletal proteins and neurite morphology are little altered at this time point (Figure 7A). However, to rule this out conclusively, we assessed Nmnat2 turnover in transected *Wld* ^S^ neurites (Figure 7C), which do not degenerate for several days. Nmnat2 levels in *Wld* ^S^ neurites fell with a remarkably similar time course to those in wild-type neurites. In contrast, cleavage of NF-H was prevented, showing that proteins that degrade as a consequence of degeneration are stabilized in *Wld* ^S^ neurites. As predicted, Wld^S^ levels in neurites also remained relatively constant. Indeed, levels of Wld^S^ protein are only moderately reduced in neurites 48 h after transection (Figure S8).

Thus, Nmnat2 is rapidly depleted in distal stumps of injured neurites, as a result of natural turnover rather than a consequence of degeneration. This is consistent with Nmnat2 loss triggering Wallerian degeneration. The continued presence of Wld^S^ in transected *Wld* ^S^ neurites long after Nmnat2 is lost shows that Wld^S^ does not act by stabilizing Nmnat2 but instead supports a model in which Wld^S^ substitutes for the functionally related Nmnat2.

### Net Anterograde Delivery of Nmnat2 by Fast Axonal Transport

We also found that an Nmnat2–enhanced green fluorescent protein (eGFP) fusion protein localizes to SCG neurites in highly defined particles shortly after being expressed (Video S1 and Figure 7D). In contrast, eGFP alone showed uniform distribution in neurites (unpublished data). Particles containing Nmnat2-eGFP travel bi-directionally, but the majority move in an anterograde direction (72.2%±3.8% based on particle movements in 18 neurites). The average and maximal velocities of particles moving anterogradely (0.58±0.09 and 1.52±0.12 µm/sec) and retrogradely (0.29±0.06 and 1.18±0.10 µm/sec) are consistent with fast axonal transport. This indicates that Nmnat2 undergoes rapid net anterograde delivery from the cell body to neurites. This is another important prediction of our model, as rapid delivery is needed to replenish constant turnover of Nmnat2 in distal neurites (above).

### Nmnat2 Protects Transected Neurites When Highly Overexpressed

Finally, if Nmnat2 is an endogenous axon survival factor, overexpression should protect transected neurites by preloading them with increased amounts of the protein. However, due to its relatively short half-life, protection should be highly dose-dependent and prolonged protection might only be achieved with very high levels of Nmnat2. In contrast, relatively long-lived Wld^S^ should also confer protection at much lower levels.

**Figure 8:**
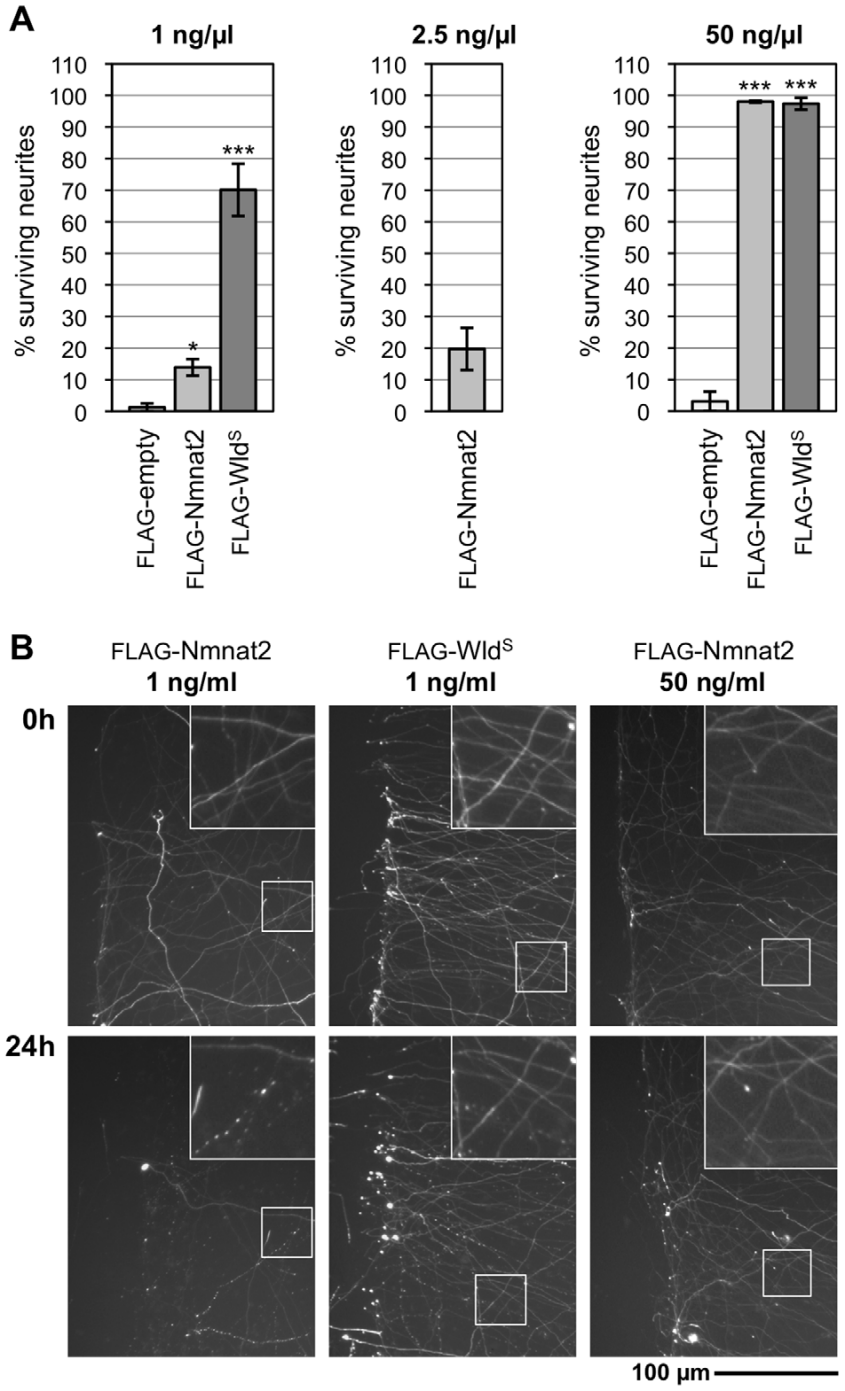
Exogenous Nmnat2 protects transected neurites for prolonged periods only when highly overexpressed. (A) Protection of transected neurites by exogenous expression of FLAG-Nmnat2 or FLAG-Wld^S^ (see also Figure S9). SCG neurons injected with 1, 2.5, or 50 ng/µl empty vector (FLAG-empty) or FLAG-Nmnat2 or FLAG-Wld^S^ expression vectors, together with pDsRed2-N1 (50 ng/µl), were transected 48 h later. Survival of DsRed2-labeled neurites 24 h after transection is shown as a percentage of the number of labelled neurites with normal morphology just after cut (0 h) and was quantified from two to four independent experiments combining data from multiple fields (error bars = ±S.E.M). FLAG-Wld^S^ protects neurites significantly better than FLAG-Nmnat2 24 h after cut at the 1 ng/µl vector concentration (***p<0.001, *t* test). There is no significant difference in protection 24 h after transection at the 50 ng/µl vector concentration. (B) Representative fluorescent images of transected DsRed2-labeled neurites of SCG neurons injected with selected concentrations of FLAG-Nmnat2 or FLAG-Wld^S^ expression vectors (as indicated). The same field of neurites is shown at the time of cut (0 h) and 24 h later. The cut site is located to the left of each image. Increased magnification of the framed region in each panel is shown for better visualization of neurite morphology.

We tested the ability of exogenous expression of tagged Nmnat2 and Wld^S^ to protect transected neurites in a microinjection-based assay (Figure S9). Dilution of the injected construct allowed controlled amounts to be reproducibly introduced into neurons. At low vector concentration (1 ng/ml), Wld^S^ conferred robust protection to neurites for 24 h after cutting, whereas Nmnat2 provided almost no protection (Figure 8A and 8B). In contrast, at 50-fold higher construct concentrations, both Nmnat2 and Wld^S^ conferred protection to almost all cut neurites at 24 h (Figure 8A and 8B). Although we used identical expression cassettes to give the best chance of equal expression of the two proteins in this assay, the shorter half-life of FLAG-Nmnat2 probably manifests as a lower steady-state level at the time of cut relative to FLAG-Wld^S^. Indeed, in transfected HEK 293T cells, we found that 2.5 times more FLAG-Nmnat2 construct was required to give steady-state protein levels approximately equal to FLAG-Wld^S^ (and the other Nmnat isoforms). Importantly, whilst we found that injection of the FLAG-Nmnat2 construct at 2.5 ng/ml gave slightly increased protection 24 h after cut relative to 1 ng/ml, this was still greatly reduced protection compared to the FLAG-Wld^S^ construct at the lower concentration (Figure 8A). Thus exogenous Nmnat2 only confers significant protection of cut neurites when expressed at high levels, consistent with its short half-life, whilst more stable Wld^S^ protects even at low levels.

## Discussion

Our results provide direct support for the hypothesis that constant delivery of a labile, cell body–synthesized survival factor is required to stop healthy mammalian axons undergoing Wallerian degeneration. Defects that prevent its delivery, including axon injury [6],[40], axonal transport impairment [27],[41], cell death [35], or disruption of protein synthesis in the cell body (Figures 1 and 2), all trigger Wld^S^-sensitive axon degeneration. We identify Nmnat2 as one such critical axon survival factor, required to maintain normal axon integrity and sufficient to preserve injured ones at high doses. Nmnat2 half-life, uniquely among the three Nmnat isoforms, is consistent with the timing of the latent phase of Wallerian degeneration and commitment to degenerate in primary culture. We also show for the first time that endogenous Nmnat2 is present in neurites, where levels drop rapidly after injury. Importantly, this is not a consequence of neurite degeneration but represents natural turnover prior to activation of degeneration. These findings have significant implications for our molecular understanding of Wallerian degeneration and “dying-back” axonopathies, and for the mechanism by which Wld^S^ and other Nmnat isoforms delay axon degeneration.

The most compelling evidence that Nmnat2 is required for maintenance of healthy axons is our observation that siRNA-mediated knock-down of Nmnat2 alone induces neurite degeneration in the absence of injury and that this precedes any effect on neuronal viability. The initiation and progression of this degeneration is clearly slower than that caused by protein synthesis suppression, but this is consistent with the mechanisms involved. The critical rate-limiting factor following translation inhibition is protein half-life, but for siRNA-mediated knock-down additional time is needed for mRNA degradation. Pharmacological inhibition of translation could also result in more efficient and homogenous knock-down. It is also possible that depletion of other axon survival factors after global suppression of protein synthesis may contribute to this difference in timing. Nmnat1 and Nmnat3 seem unlikely to be among them in this experimental system because of their long half-lives, the absence of any clear effect of their siRNAs, and the fact that endogenous levels of both proteins cannot compensate for loss of Nmnat2.

Nmnat2 is a labile protein in HEK cells, in whole SCG explants, and in transected neurites. The rate of Nmnat2 turnover is consistent with the trigger for axon degeneration being depletion below a critical threshold. Nmnat2 falls to barely detectable levels in transected wild-type SCG neurites prior to any significant physical signs of degeneration, which suggests that the critical threshold level of Nmnat2 is quite low. However, the precise threshold level is difficult to determine because the duration of downstream steps needed to bring about degeneration is unknown. Steady-state levels of Nmnat2 in SCG neurites also seem quite low and this could account for the short latent phase between neurite transection and degeneration in these cultures.

Of the three mammalian Nmnat isoforms, Nmnat2 did not initially appear the most obvious candidate for an endogenous axon survival factor, despite being the most abundantly expressed isoform in the nervous system at the mRNA level [23],[34]. First, its predominant Golgi localization seemed inconsistent with an axonal location. However, a recent report shows axons in primary neuronal cultures contain Golgi components [42] and, as with Wld^S^ [3], predominant localization may not reflect the site of its axon protective role. We have now clearly detected endogenous Nmnat2 in SCG neurites by immunoblotting and have shown that an Nmnat2-eGFP fusion localizes to distinct, rapidly transported particles in these neurites (Figure 7 and Video S1). It will be interesting to determine the precise nature of these particles. Second, the inability of Nmnat2 to protect 5-d lesioned axons in *Drosophila*, unlike the other Nmnat isoforms and Wld^S^, initially suggested it was either ineffective or by far the least potent isoform [2]. However, it has more recently been shown that exogenous expression of Nmnat2 can protect injured mammalian axons [26]. We propose that the short half-life of Nmnat2 could provide an explanation for this discrepancy, with the degree of protection being related to the levels of Nmnat2 expression achieved in the different systems. It is also possible that some protection of lesioned *Drosophila* axons might be evident at a less stringent time point (wild-type fly axons begin to degenerate just 1 d after injury). Thus, a short half-life, one of the most critical inherent properties of the endogenous survival factor in our model, might mask the capacity of exogenous Nmnat2 to protect in some situations. Conversely, greater stability probably underlies the ability of exogenous Nmnat1 and Nmnat3 to protect injured axons/neurites more robustly in this and other in vivo and/or in vitro situations [1],[2],[8],[25].

Our data suggest that Wld^S^ may protect axons by directly substituting for loss of endogenous Nmnat2 after injury or other stresses. This is based on three principal observations. First, Wld^S^ is inherently more stable than Nmnat2, decaying less in 48 h than Nmnat2 does in 4 h after neurite transection (Figures 6, 7, and S8). Importantly, continued degradation of Nmnat2 in *Wld* ^S^ neurites rules out an alternative hypothesis, that Wld^S^ could delay axon degeneration by stabilizing Nmnat2. Second, Wld^S^ and Nmnat2 share the same enzyme activity, which is required for their ability to protect axons [5],[26],[43]. Third, both are present in axons (Figure 7, Video S1, and [3]), and the presence of Wld^S^ in microsome fractions [3],[8] is consistent with a possible shared localization with Nmnat2 in Golgi, or Golgi-derived structures in axons [42].

The ability of exogenous nuclear Nmnat1 and mitochondrial Nmnat3 to confer axon protection in a number of situations outwardly seems to contradict the claim that Nmnat localization is actually important, but there is increasing evidence to support it. First, endogenous Nmnat1 and Nmnat3 (which do appear to be expressed in SCG neurons; Figure S3) cannot compensate for loss of Nmnat2 (Figure 4), probably as a result of strict compartmentalization. Alternatively, this could simply reflect the relative contributions of each isoform to total basal Nmnat activity in these axons. Second, redistribution of predominantly nuclear Wld^S^ and Nmnat1 into the cell body and axon enhances their ability to delay Wallerian degeneration [3],[7],[8]. Finally, Nmnat1 and Nmnat3 only confer protection when overexpressed. This appears to be accompanied by significant mis-localization (Figure 3B, unpublished observations, and [8]), which may cause a serendipitous increase in effective Nmnat levels in the relevant axonal location. The ability of barely detectable extra-nuclear Wld^S^ to protect injured *Wld* ^S^ mouse axons suggests that minimal mis-localization of relatively stable Nmnat1 and Nmnat3 may be sufficient to confer strong protection. The absence of significant axon protection in transgenic mice expressing Nmnat1 in neurons at similar levels to Wld^S^ in *Wld* ^S^ neurons [4],[8] suggests either that Nmnat1 localization is more rigorously controlled in vivo or that Nmnat1 overexpression is greater in vitro. Importantly, Nmnat1 can only protect mammalian axons in vivo when specifically mutated to cause mis-localization [7].

The main known function of the mammalian Nmnat isoforms is NAD^+^ biosynthesis, and the ability of Nmnat1, Nmnat2, and Wld^S^ to delay Wallerian degeneration requires Nmnat enzyme activity [1],[5],[26],[43]. NAD^+^ production may therefore underlie the ability of endogenous Nmnat2 to prevent spontaneous axon degeneration. However, there is much disagreement over the ability of NAD^+^ to protect axons directly [1],[4],[8],[44],[45], or even its involvement at all [43]. Indeed, siRNA-mediated knock-down of Nampt, the rate-limiting enzyme upstream of Nmnat in the NAD^+^ salvage pathway, does not itself trigger axon degeneration despite a substantial 70%–90% reduction in NAD^+^ levels, leading to the suggestion that an alternative Nmnat metabolite may be involved [43].

Regarding downstream events, the rapid initiation and progression of Wallerian degeneration is more consistent with an active degeneration program than passive degeneration resulting simply from loss of an essential metabolic activity. Recently, dual leucine kinase (DLK) and JNK signalling have been implicated in regulating Wallerian degeneration of DRG neurites [46]. We have found the same JNK inhibitor (SP600125) used in that study also significantly delays Wallerian degeneration of SCG neurites (unpublished data). The localization of Nmnat2 in defined particles in axons and the role it plays in them could now be key to identifying additional downstream events.

Whilst neurite degeneration in primary neuronal cultures is a useful model of in vivo axon degeneration, high levels of protein overexpression can give misleading outcomes (discussed above) and other differences need to be considered. For example, there is a much longer latent phase before fragmentation of axons in transected sciatic nerves in vivo (36–40 h [15]) than for transected SCG neurites in culture (∼8 h). This could reflect differences in the half-life of Nmnat2 in vivo and in culture (for which there is some precedent [47]), steady-state levels of Nmnat2, or the involvement of additional factors that are more critical for axon survival in vivo. However, it would be somewhat surprising if Nmnat2 did not play a critical role in vivo based on its rapid turnover and it being required for neurite survival in vitro. Other Nmnat isoforms remain candidates in vivo, particularly Nmnat3 as its mitochondrial localization makes its presence in axons likely. Indeed, a contribution from other molecules could help to explain the longer latent phase in vivo.

It will also be interesting to see whether endogenous Nmnat proteins are involved in axon survival in non-mammalian organisms such as *Drosophila*. Whilst loss of the single *Drosophila Nmnat* homolog causes degeneration of photoreceptors, this appears to be a more general effect on neuronal viability, rather than axon health, and does not require its NAD^+^-synthesizing activity [48]. This contrasts with the protection against axon degeneration by mammalian Nmnat isoforms and Wld^S^, which does require enzyme activity [1],[5],[26],[43]. Neuronal viability could therefore be dependent on a reported Nmnat-associated chaperone activity [49], with axons having a more specific dependency on enzyme activity. Thus, it is possible that the small decrease in neuron survival associated with Nmnat2 knock-down in SCG neurons (Figure 5A) could be due to loss of chaperone activity, although it is not yet known whether Nmnat2 possesses this activity like the other mammalian isoforms [49].

Loss of Nmnat2 could also underlie “dying-back” axon degeneration in disease. Due to its rapid turnover, Nmnat2 might fail to reach distal axons in sufficient quantities when axonal transport is either pathologically compromised [12],[27],[50] or slows during normal ageing [51]. Impairment of protein synthesis would be predicted to have a similar outcome, which could explain axon degeneration accompanying viral infections as the cellular protein synthesis machinery is overwhelmed [52]. More subtle effects on protein synthesis resulting from cell body defects, such as vacuolization, could underlie Wallerian-like “dying-back” axon degeneration and/or neuromuscular junction loss in slowly developing, chronic diseases like ALS [53],[54], even in the absence of neuronal loss. Our model would additionally explain why the longest axons are often most susceptible in disease. The ability of some larger mammals to support very long axons (up to several meters in some cases) raises the intriguing possibilities that Nmnat2 is inherently more stable in larger species or that chaperones stabilize it during transport.

We propose that increasing Nmnat2 stability or its delivery to axons could have important therapeutic implications for these and other disorders characterized by Wallerian-like degeneration. Both treatments should delay the point at which axons become committed to degenerate (like Wld^S^). Such therapies might be particularly effective when axonopathy results from a short-term impairment (e.g., of cell body metabolism, axonal transport, glial support, etc.) lasting just a few hours to a few weeks. Examples include Taxol-induced neuropathy, relapsing-remitting multiple sclerosis, some viral disorders, and stroke. Axons could be saved permanently if the degeneration commitment point is delayed long enough for the causative defect to be removed or to abate naturally. Although *Wld* ^S^ mice have already been shown to be resistant to Taxol-induced neuropathy [41], developing therapies based on the Wld^S^ neuroprotective mechanism has been limited by the technical challenge of introducing exogenous Wld^S^ (or other stable Nmnat isoforms). In contrast, pharmacological manipulation of endogenous Nmnat2 should be more feasible.

Finally, the increase in Nmnat2 levels in SCG cell bodies/proximal neurite stumps that we observed shortly after transection of their neurites is also intriguing (Figure 7A). The simplest explanation is that this represents accumulation of Nmnat2 in a reduced cellular volume following neurite removal while synthesis continues at pre-injury levels. However, the possibility that this could represent a stress response cannot be completely excluded at this time, especially in light of the recent report that the *Drosophila* Nmnat isoform can act as a chaperone [49]. Irrespective of the mechanism involved, this increase in Nmnat2 levels might nevertheless facilitate subsequent neurite regeneration.

In summary, we propose a model in which sustained expression and anterograde delivery of Nmnat2 is required to prevent activation of an intrinsic axon degeneration program. Degeneration is triggered when synthesis and/or delivery of Nmnat2 is disrupted and rapid turnover causes its level to drop below a critical threshold. We additionally propose that the relatively stable Wld^S^ fusion protein delays axon degeneration by directly substituting for loss of Nmnat2 and that localization may be an important factor. Endogenous Nmnat2 represents an exciting target for therapeutic manipulation.

## Materials and Methods

### Plasmids Constructs and siRNA Reagents

Expression vectors encoding FLAG-tagged murine Nmnat isoforms and Wld^S^ were generated by amplification of the full coding region of each gene by Reverse Transcriptase PCR (RT-PCR) (see below) from 1 µg total RNA from wild-type and *Wld^S^* mouse brain. Products were cloned into pCMV Tag-2B (Stratagene) to generate FLAG-Nmnat/Wld^S^ expression vectors or pEGFP-N1 (BD Biosciences Clontech) to generate a Nmnat2-eGFP expression vector. Sequencing (Cogenics) was performed to confirm the absence of PCR errors. Other plasmids used were pDsRed2-N1 for expression of variant *Discosoma* red fluorescent protein (DsRed2) and pEGFP-C1 for expression of eGFP (both BD Biosciences Clontech). Dharmacon ON-TARGET*plus* SMART pools of siRNA (Thermo Scientific) specifically targeted against mouse *Nmnat1* (L-051136-01), *Nmnat2* (L-059190-01), or *Nmnat3* (L-051688-01) were used in this study. Dharmacon ON-TARGET*plus* si*Control* non-targeting siRNA pool (D-001810-10) was used as a control in experiments. Each pool consists of 4 individual siRNAs. The siRNAs making up the ON-TARGET*plus Nmnat2* SMART pool (J-059190-09, -10, -11, and -12) were also tested individually or in subpools.

### Cell Culture

#### Explant cultures

SCGs were dissected from P1 or P2 mouse or rat pups, and DRGs were dissected from E15.5 mouse embryos. Cleaned explants were placed in the centre of 3.5 cm tissue culture dishes pre-coated with poly-L-lysine (20 µg/ml for 1–2 h; Sigma) and laminin (20 µg/ml for 1–2 h; Sigma). Explants were cultured in Dulbecco's Modified Eagle's Medium (DMEM) with 4,500 mg/L glucose and 110 mg/L sodium pyruvate (Sigma), 2 mM glutamine, 1% penicillin/streptomycin, 100 ng/ml 7S NGF (all Invitrogen), and 10% fetal bovine serum (Sigma). Four µM aphidicolin (Calbiochem) was used to reduce proliferation and viability of small numbers of non-neuronal cells. Cultures were used after 5–7 d.

#### Dissociated SCG cultures

Dissected SCG ganglia were incubated in 0.025% trypsin (Sigma) in PBS (without CaCl_2_ and MgCl_2_) for 30 min followed by 0.2% collagenase type II (Gibco) in PBS for 30 min. Ganglia were then gently triturated using a pipette. After a 2-h pre-plating stage to remove non-neuronal cells, 5–10,000 dissociated neurons were plated in a 1 cm^2^ poly-L-lysine and laminin-coated area of normal 3.5 cm dishes (Nunc) or ibidi μ-dishes (Thistle Scientific) for microinjection experiments. Dissociated cultures were maintained as explant cultures except that 20 µM uridine and fluorodeoxyuridine was used to reduce proliferation and viability of non-neuronal cells (Sigma).

#### Compartmented cultures

Dissected SCG explants were broken into small pieces using forceps and then placed into the central compartment of three-compartment Campenot Teflon divider (Tyler Research) essentially as described previously [55]. The ability of the barriers to prevent diffusion of bromophenol blue between the independent compartments containing the cell bodies and distal neurites for at least 24 h after completion of the experiment was assessed to confirm their integrity. Compartmented cultures were maintained as explant cultures.

#### HEK 293 culture

HEK 293 cells were cultured under standard conditions in DMEM with 4,500 mg/L glucose and 110 mg/L sodium pyruvate (PAA), supplemented with 2 mM glutamine and 1% penicillin/streptomycin (both Invitrogen), and 10% fetal bovine serum (Sigma).

#### Animals

C57BL/6JOlaHsd and homozygous C57BL/6OlaHsd-Wld (*Wld* ^S^) mice and Sprague Dawley rats were obtained from Harlan UK (Bicester, UK). Transgenic *Wld* ^S^ rat line 79 has been described previously [56]. All animal work was carried out in accordance with the Animals (Scientific Procedures) Act, 1986, under Project Licenses PPL 80/1778 and 80/2254.

### RT-PCR

Total brain RNA was extracted using TRIzol reagent (Invitrogen), and RNA from dissociated SCG neuronal cultures was isolated using RNeasy columns (Qiagen). One µg of brain RNA and 30% of that recovered from SCG cultures was reverse transcribed into cDNA using Superscript II (both Invitrogen). Control samples without reverse transcriptase were processed simultaneously to rule out DNA contamination of samples. Standard PCR amplification was performed using REDTaq DNA polymerase (Sigma). Primers used for detection of *Nmnat* isoform transcripts in SCG neuron RNA were as follows: *Nmnat1* 5′-ttcaaggcctgacaacatcgc-3′ and 5′-gagcaccttcacagtctccacc-3′, *Nmnat2* 5′-cagtgcgagagacctcatccc-3′ and 5′-acacatgatgagacggtgccg-3′, *Nmnat3* 5′-ggtgtggaggtgtgtgacagc-3′ and 5′-gccatggccactcggtgatgg-3′. Products were sequenced to confirm correct amplification.

### Inhibitor Treatments

1,000× aqueous stock solutions of emetine (dihydrochloride hydrate) and CHX in DMSO (both Sigma) were diluted 1∶1000 in culture media to give final concentrations indicated (1 µg/ml CHX = 3.5 µM). InSolution MG-132 (Calbiochem) was diluted to 20 µM. MG-132 was added to SCG explant cultures 3 h prior to neurite transection. This pre-treatment is required to see neurite protection in these cultures [18]. Media was changed once with addition of fresh inhibitors when cultures were treated for more than 5 d. CHX-containing media was completely removed and replaced with media containing no CHX in experiments involving temporary suppression of protein synthesis.

### Microinjection and Immunostaining

Microinjection was performed on a Zeiss Axiovert 200 microscope with an Eppendorf 5171 transjector and 5246 micromanipulator system and Eppendorf Femtotips. Plasmids and siRNAs were diluted in 0.5× PBS and passed through a Spin-X filter (Costar). The mix was injected directly into the nuclei of SCG neurons in dissociated cultures. ON-TARGET*plus* siRNA pools were injected at a concentration of 100 ng/µl and individual siRNAs or sub-pools as indicated in the text, pDsRed2-N1 at 50 ng/µl, pEGFP-C1 at 10 ng/µl, the Nmnat2-eGFP expression construct at 50 ng/µl, and FLAG-Nmnat/Wld^S^ expression constructs or FLAG-empty control (pCMV Tag-2B) at 10 ng/µl for siRNA-mediated knock-down analysis by immunostaining (Figure 3B) and at 1, 2.5 or 50 ng/µl in neurite transection experiments (Figure 8 and Figure S9). Seventy to 150 neurons were injected per dish. Injection of relatively few neurons per dish facilitated visualization of individual labelled neurites as neurites tend to cluster together in bundles. For detection of FLAG-tagged protein expression by immunostaining, neurons were fixed with 4% paraformaldehyde (20 min), permeabilized with 1% Triton X-100 in PBS (10 min), blocked in 50% goat serum in PBS containing 1% BSA (30 min), and stained using monoclonal M2 anti-FLAG (Sigma) (1∶400 in PBS, 1% BSA for 1 h) and an Alexa568-conjugated secondary antibody (1∶200 in PBS, 1% BSA for 1 h). Cells were mounted in Vectashield containing DAPI (Vector Laboratories) for counterstaining of nuclei. For comparing the quantification of neuronal viability based on gross morphology with other indicators of health (Figure 5B), cultures were incubated with 1 µg/ml propidium iodide (Invitrogen) for 15 min and were then fixed with 4% paraformaldehyde (20 min) before being mounted in Vectashield containing DAPI.

### Neurite Transection for Imaging and Quantification of Degeneration

Neurites were cut with a disposable scalpel roughly half-way between their cell bodies and their most distal ends. Where applicable, inhibitors of translation or vehicle (DMSO) were added to the media less than 10 min before transection. Uncut neurites treated with DMSO continue to grow normally (unpublished data). Microinjection of a row of cell bodies in dissociated SCG cultures enabled neurites to be cut so that all injected cell bodies and their proximal neurites were located on the opposite side of the cut site to the distal stumps (Figure S9).

### Western Blot Analysis

#### HEK 293 transfection

Cells were plated so that they reached 60%–80% confluence on the day of transfection and were transfected using Lipofectamine 2000 reagent (Invitrogen). For turnover experiments (Figure 6), cells in a 12-well dish format were co-transfected with 100 ng each of the FLAG-Nmnat1, FLAG-Nmnat-3, and FLAG-Wld^S^ expression constructs and 250 ng of the FLAG-Nmnat-2 expression construct. For specificity experiments (Figure 3), 1 µg of one of the siRNA pools was also added as indicated. After the treatments described in the text, cells were lysed directly into 2× Laemmli sample buffer after washing with PBS.

#### SCG neurite extract preparation

Following treatment (as indicated), ganglia in 6- or 7-d-old SCG explant cultures were separated from their neurites with a scalpel. Ganglia (including proximal neurite stumps) and neurites were collected separately, washed in PBS, and lysed and processed as above.

#### Immunoblotting

Extracts were separated by standard SDS-PAGE on SDS polyacrylamide gels (6%–13% depending on the proteins being detected) and transferred to Immobilon-P membrane (Millipore) using the Bio-Rad Mini-PROTEAN III wet transfer system. Blots were blocked and incubated with primary antibodies overnight (in 1× TBS p.H. 8.3, with 0.05% Tween 20 and 5% milk powder or 5% BSA) followed by the appropriate HRP-conjugated secondary antibody (1 h at 1∶2000–1∶5000 dilution) and detection by ECL (Amersham Pharmacia Biotech) with washes between each stage. The following primary antibodies were used: mouse monoclonal anti-FLAG (1∶2000–1∶5000, Sigma, M2), rabbit polyclonal anti-Wld^S^ (1∶4000, Wld18), mouse monoclonal anti-NF-H (1∶2000, Sigma, N52), mouse monoclonal anti-Nmnat2 (2.0 µg/ml, Abcam, ab56980), mouse monoclonal anti-neuronal class βIII-Tubulin (1∶2000–1∶10,000, Covance, MMS-435P), and mouse monoclonal anti-Histones (1∶1000, Millipore, MAB052). In Figures 6C, 7A, 7B, and 7C, exactly 10% of the extract that was used for the blot probed with the Nmnat2 antibody (and Wld^S^ and Histones antibodies where applicable) was used for the blot probed with the β-Tubulin antibody (and NF-H antibody where applicable) to avoid signal saturation. Relative band intensities on scanned autoradiographs were determined using ImageJ software version 1.43 (NIH, Bethesda, Maryland, USA, http://rsb.info.nih.gov/ij/). Statistical analysis was performed using a two-tailed *t* test.

### Microscopy and Imaging

Bright-field and fluorescence images were captured on an Olympus IX81 inverted fluorescence microscope using a Soft Imaging Systems F-View camera linked to a PC running the appropriate imaging software. Wherever possible, images of the same field of neurites or neuronal cell bodies were captured at the indicated time points after initial manipulation. Images were processed using Adobe Photoshop Elements 4.0. The intensity of FLAG immunostaining relative to eGFP fluorescence in individual injected neurons (Figure 3) was quantified using ImageJ software. Images were captured for analysis using identical microscope settings between samples for each channel. Time-lapse images of Nmnat2-eGFP transport were acquired 6 h after injection of the expression vector using an Olympus Cell^R^ imaging system comprising IX81 microscope linked to a Hamamatsu ORCA ER camera and a 100×1.45 NA apochromat objective. Cultures were maintained at 37°C in a Solent Scientific environment chamber. Wide-field epifluorescence images were captured at 2 Hz for 1 min. ImageJ software plug-ins were used for analysis of the stacks (kymograph generation and analysis of particle velocities) and conversion of an image stack into an annotated movie (Video S1).

### Quantification of Neurite Degeneration

#### Neurite blebbing

Membrane blebs more than twice the width of the associated neurite or neurite bundle were counted in a 100×100 µm box in bright-field images of the same neurites just after treatment (0 h) and the indicated times afterwards. Bleb numbers are likely to be under-scored on highly degenerated neurites due to clustering of multiple blebs that cannot easily be individually differentiated or fragment loss. Statistical analysis was performed using a two-tailed *t* test.

#### Degeneration of fluorescent marker-labelled neurites. 

Numbers of morphologically normal and continuous Ds-Red2- or eGFP-labelled neurites were counted in the same field of distal neurites at various times after manipulation/injection. In siRNA injection experiments (Figures 4 and 5) and transection experiments (Figure 8), the percentage of healthy neurites remaining relative to the first time point was determined. Neurites were deemed unhealthy if they displayed abnormal morphology (including those with multiple swellings) or had undergone fragmentation. Neurite outgrowth still occurred from neurons injected with si*Control*, si*Nmnat1*, or si*Nmnat3*, but any neurites that grew into the analyzed field during the time course were not counted. In each case statistical analysis was performed using a two-tailed *t* test.

## Supporting Information

Figure S1 **Suppression of protein synthesis induces Wallerian-like neurite degeneration in rat SCG and mouse DRG explant cultures.** Representative bright-field images of distal neurites in (A) wild-type (Sprague Dawley) or *Wld* ^S^ transgenic rat SCG explant cultures and (B) wild-type or *Wld* ^S^ mouse DRG explant cultures each treated with 10 µM emetine. Images were captured at the indicated times after emetine addition and are representative of multiple fields in three independent experiments. DRG neurites appear to be more resistant than SCG neurites to the effects of translation inhibitors as 20 µg/ml CHX was required to consistently induce neurite degeneration by 24 h (unpublished data). This is consistent with DRG neurites also undergoing Wallerian degeneration at a slower rate than SCG neurites (compare Figure S2B with [25]).
(1.78 MB TIF)

Figure S2 **Neither** ***Wld*** **^S^-mediated protection of transected SCG neurites nor Wallerian degeneration itself requires localized translation in neurites.** Representative bright-field images of transected neurites from (A) *Wld* ^S^ or (B) wild-type (BL/6) mouse SCG explant cultures treated with vehicle (untreated), 1 µg/ml CHX, or 10 µM emetine. Inhibitors were added just before transection and images captured at the times indicated after cut. Part of the cut site is visible in the bottom left-hand corner. Increased magnification of framed regions is shown for better visualization of neurites. Healthy-looking *Wld* ^S^ neurites occasionally detached from the culture dish prior to 72 h after cut probably due to handling of the cultures. Images are representative of multiple fields in two or more independent experiments.
(4.79 MB TIF)

Figure S3 **RT-PCR analysis indicates that mRNAs of all three** ***Nmnat*** **isoforms are expressed in SCG neurons.** Amplification products were resolved on a 2% ethidium bromide-stained agarose gel. The image is representative of three independent experiments. Reactions with no reverse transcriptase (no RT) were included to confirm no DNA contamination. The amplification kinetics of each set of primers are approximately equivalent (unpublished data), suggesting that *Nmnat2* mRNA may be expressed at slightly higher levels than *Nmnat1* and *Nmnat3* mRNAs.
(0.07 MB TIF)

Figure S4 **Microinjection-based strategy for assessing the effects of siRNAs in SCG neurons.** Nuclei were injected with a mix containing siRNA (here injected with si*Control*) and pDsRed2-N1 (50 ng/µl). High expression of DsRed2 allowed clear visualization of injected neurons and complete neurite projections 24 h after injection. The intensity of DsRed2-labeling increases over subsequent days. A line of neuronal cell bodies was injected to simplify differentiation of the proximal and distal portions of neurites. Distal neurites were imaged to allow their health to be monitored 24, 48, and 72 h after injection.
(0.37 MB TIF)

Figure S5 **Neurite degeneration triggered by** ***Nmnat2*** **siRNA is a result of Nmnat2 knock-down rather than an off-target effect.** (A) Effects of the four siRNAs making up the si*Nmnat2* pool – J-059190-09, -10, -11, and -12—injected individually or in subpools (together with pDsRed2-N1 at 50 ng/µl). Neurite survival is shown as a percentage of the total number of DsRed2-labeled neurites (healthy and abnormal) at 24 h and was quantified from three independent experiments combining data from multiple fields (error bars = ±S.E.M.). J-059190-11 alone (100 ng/µl) and J-059190-10 and J-059190-12 in combination (50 ng/µl each) caused significant loss of distal neurites comparable to the si*Nmnat2* pool (**p<0.01, ***p<0.001, *t* test versus equivalent time point for si*Control*). J-059190-09, -10, and -12 caused only limited neurite degeneration individually (unpublished data). J-059190-09 injected at 25 ng/µl, equivalent to its contribution in the si*Nmnat2* pool (at 100 ng/µl), also caused significant neurite loss (*p<0.05, *t* test versus equivalent time point for si*Control*), but this was significantly reduced compared to injection at 100 ng/µl (^§^ *p*<0.05, ^§§^ *p*<0.01, *t* test J-059190-11 at 25 ng/µl versus 100 ng/µl at equivalent time points). (B) Representative fluorescent images of DsRed2-labeled neurites of wild-type (BL/6) SCG neurons 24 and 72 h after injection with J-059190-11 or J-059190-10+-12 (together with pDsRed2-N1). Abnormal neurite morphology and neurite loss at 72 h is identical to that seen following injection of the si*Nmnat2* pool (Figure 4).
(0.56 MB TIF)

Figure S6 **Loss of neuron viability after injection with siRNA and the DsRed2 expression vector at high concentration.** (A–D) Quantification of neuron viability matched to the analyses of neurite survival in Figure 4 and Figure S5 as indicated. The number of neurons with normal gross morphology remaining at each time point is shown as a percentage of those present at 24 h (error bars = ±S.E.M.). Injection of si*Nmnat2* appeared to cause a decrease in neuronal viability relative to si*Control* in some experiments (A–C) and this was prevented in *Wld* ^S^ neurons (C). Individual siRNAs from the si*Nmnat2* pool had equivalent effects on neuronal viability (D). (E) Neuron viability was scored based on gross morphology (in bright-field and DsRed2 imaging) of the injected neuron cell bodies. Two of the three injected neurons shown with normal morphology in the top panel appear abnormal 24 h later (arrowheads, bottom panel). Assessment based on gross morphology closely matches other indicators of cell viability (see Figure 5B). (F) Comparison between the amounts of neuron and neurite loss induced by si*Nmnat2*. Percentages of viable neurons and healthy neurites at each time point after si*Nmnat2* injection were normalized to matched si*Control* percentages in all paired experiments performed (*n* = 8) and are expressed as a fraction of si*Control* values (error bars = ±S.E.M). The significant reduction in neurite health following injection of si*Nmnat2* is proportionately far greater than the small but significant reduction in neuronal viability (*p<0.05, ***p<0.001, *t* test si*Nmnat2* versus si*Control* at equivalent time points; ^§§§^ *p*<0.001, *t* test si*Nmnat2* neurite survival versus si*Nmnat2* neuron survival at equivalent time points). In contrast, neuron loss exceeds neurite degeneration with all other siRNA pools. This is presumably due to preferential loss of neurons that do not project neurites into areas where degeneration is assessed. This may be because neurons with shorter neurites are more susceptible to the DsRed2 toxicity that causes background cell death in these experiments (see main text).
(0.83 MB TIF)

Figure S7 **Wild-type SCG neurites become committed to degenerate after approximately 6 h of protein synthesis suppression and this is delayed by Wld^S^.** Representative bright-field images of distal neurites from (A) wild-type (BL/6) or (B) *Wld* ^S^ mouse SCG explant cultures treated with 1 µg/ml CHX for 4, 8, or 24 h before inhibitor removal. Images of the same field of neurites were captured at the indicated times after initial addition of the inhibitor (0 h). Framed regions are magnified for better visualization of neurite morphology. A dashed white line provides a reference point against which relative neurite extension can be assessed. Images for the 4 h and 8 h treatments of BL/6 cultures are representative of nine fields in three independent experiments, and images for the 24 h treatment of *Wld* ^S^ cultures are representative of 8 out of 10 fields in four independent experiments. An intermediate 6 h treatment of BL/6 neurites resulted in a mixed outcome; neurites became significantly blebbed by 24 h in four out of nine fields in three independent experiments. A 48 h treatment of *Wld* ^S^ cultures blocked neurite outgrowth in all six fields in three independent experiments and caused blebbing by day 9 in four out of six fields (unpublished data).
(2.96 MB TIF)

Figure S8 **Wld^S^ levels are only slightly reduced in** ***Wld*** **^S^ neurites 48 h after transection.** Representative immunoblot showing levels of Wld^S^ in transected *Wld* ^S^ neurites just after cut (0 h) and 48 h later. ß-Tubulin acts as a loading control. Absence of the 16 kDa core Histones band in neurite extracts confirms there is no detectable contamination with SCG cell bodies or non-neuronal cells. Each lane represents material collected from SCG explant cultures derived from 8–10 ganglia (below the threshold for consistent detection of Nmnat2). Wld^S^ band intensity at 48 h is plotted (centre) as a fraction of that at 0 h after normalization to ß-Tubulin and was quantified from three independent experiments (error bars = ±S.E.M.). Images of the same field of neurites (right) show representative transected neurite morphology at 0 h and 48 h.
(0.36 MB TIF)

Figure S9 **Assessing injury-induced degeneration of DsRed2-labeled SCG neurites.** Representative fluorescent images of the same field of transected DsRed2-labeled neurites of wild-type (BL/6) neurons injected with 1 ng/µl empty FLAG vector (FLAG-empty) and pDsRed2-N1 (50 ng/µl) immediately after cut (0 h) and 24 h later. Lower magnification images show a line of injected neuronal cell bodies and the neurite network projecting from them. This pattern of injection facilitated transection such that all injected neurons and their proximal neurites were located on the opposite side of the cut to the transected distal neurites. The location of the cut site is indicated. All neurites disconnected from their cell bodies have degenerated by 24 h. Identical results were obtained with 50 ng/µl FLAG-empty. Increased magnification of the framed regions is shown for better visualization of neurites.
(0.85 MB TIF)

Video S1 **Time-lapse imaging of Nmnat2-eGFP transport in SCG neurites.** SCG neurons were injected with the Nmnat2-eGFP expression vector and time-lapse images captured 6 h later. The movie shows several neurites of injected neurons that were located to the left of the imaged field. The movie is speeded up 5× and represents 1 min in real time. A counter (in s) is shown at the top right.
(4.67 MB AVI)

## Abbreviations

CHX: cycloheximide
DRG: dorsal root ganglion
eGFP: enhanced green fluorescent protein
NF-H: neurofilament heavy chain
RT-PCR: Reverse Transcriptase PCR
SCG: superior cervical ganglion

## References

[1] Araki T, Sasaki Y, Milbrandt J (2004). Increased nuclear NAD biosynthesis and SIRT1 activation prevent axonal degeneration.. Science.

[2] Avery M. A, Sheehan A. E, Kerr K. S, Wang J, Freeman M. R (2009). Wld^S^ requires Nmnat1 enzymatic activity and N16-VCP interactions to suppress Wallerian degeneration.. J Cell Biol.

[3] Beirowski B, Babetto E, Gilley J, Mazzola F, Conforti L (2009). Non-nuclear Wld^S^ determines its neuroprotective efficacy for axons and synapses in vivo.. J Neurosci.

[4] Conforti L, Fang G, Beirowski B, Wang M. S, Sorci L (2007). NAD(+) and axon degeneration revisited: Nmnat1 cannot substitute for Wld^S^ to delay Wallerian degeneration.. Cell Death Differ.

[5] Conforti L, Wilbrey A, Morreale G, Janeckova L, Beirowski B (2009). Wld^S^ protein requires Nmnat activity and a short N-terminal sequence to protect axons in mice.. J Cell Biol.

[6] Mack T. G, Reiner M, Beirowski B, Mi W, Emanuelli M (2001). Wallerian degeneration of injured axons and synapses is delayed by a Ube4b/Nmnat chimeric gene.. Nat Neurosci.

[7] Sasaki Y, Vohra B. P, Baloh R. H, Milbrandt J (2009). Transgenic mice expressing the Nmnat1 protein manifest robust delay in axonal degeneration in vivo.. J Neurosci.

[8] Yahata N, Yuasa S, Araki T (2009). Nicotinamide mononucleotide adenylyltransferase expression in mitochondrial matrix delays Wallerian degeneration.. J Neurosci.

[9] Chevalier-Larsen E, Holzbaur E. L (2006). Axonal transport and neurodegenerative disease.. Biochim Biophys Acta.

[10] Hafezparast M, Klocke R, Ruhrberg C, Marquardt A, Ahmad-Annuar A (2003). Mutations in dynein link motor neuron degeneration to defects in retrograde transport.. Science.

[11] Puls I, Jonnakuty C, LaMonte B. H, Holzbaur E. L, Tokito M (2003). Mutant dynactin in motor neuron disease.. Nat Genet.

[12] Reid E, Kloos M, Ashley-Koch A, Hughes L, Bevan S (2002). A kinesin heavy chain (KIF5A) mutation in hereditary spastic paraplegia (SPG10).. Am J Hum Genet.

[13] Zhao C, Takita J, Tanaka Y, Setou M, Nakagawa T (2001). Charcot-Marie-Tooth disease type 2A caused by mutation in a microtubule motor KIF1Bbeta.. Cell.

[14] Lubinska L (1977). Early course of Wallerian degeneration in myelinated fibres of the rat phrenic nerve.. Brain Res.

[15] Beirowski B, Berek L, Adalbert R, Wagner D, Grumme D. S (2004). Quantitative and qualitative analysis of Wallerian degeneration using restricted axonal labelling in YFP-H mice.. J Neurosci Methods.

[16] MacInnis B. L, Campenot R. B (2005). Regulation of Wallerian degeneration and nerve growth factor withdrawal-induced pruning of axons of sympathetic neurons by the proteasome and the MEK/Erk pathway.. Mol Cell Neurosci.

[17] Tsao J. W, George E. B, Griffin J. W (1999). Temperature modulation reveals three distinct stages of Wallerian degeneration.. J Neurosci.

[18] Zhai Q, Wang J, Kim A, Liu Q, Watts R (2003). Involvement of the ubiquitin-proteasome system in the early stages of Wallerian degeneration.. Neuron.

[19] Beirowski B, Adalbert R, Wagner D, Grumme D. S, Addicks K (2005). The progressive nature of Wallerian degeneration in wild-type and slow Wallerian degeneration (*Wld*
^S^) nerves.. BMC Neurosci.

[20] Kerschensteiner M, Schwab M. E, Lichtman J. W, Misgeld T (2005). *In vivo* imaging of axonal degeneration and regeneration in the injured spinal cord.. Nat Med.

[21] Conforti L, Tarlton A, Mack T. G, Mi W, Buckmaster E. A (2000). A Ufd2/D4Cole1e chimeric protein and overexpression of Rbp7 in the slow Wallerian degeneration (*Wld*
^S^) mouse.. Proc Natl Acad Sci U S A.

[22] Emanuelli M, Carnevali F, Saccucci F, Pierella F, Amici A (2001). Molecular cloning, chromosomal localization, tissue mRNA levels, bacterial expression, and enzymatic properties of human NMN adenylyltransferase.. J Biol Chem.

[23] Raffaelli N, Sorci L, Amici A, Emanuelli M, Mazzola F (2002). Identification of a novel human nicotinamide mononucleotide adenylyltransferase.. Biochem Biophys Res Commun.

[24] Zhang X, Kurnasov O. V, Karthikeyan S, Grishin N. V, Osterman A. L (2003). Structural characterization of a human cytosolic NMN/NaMN adenylyltransferase and implication in human NAD biosynthesis.. J Biol Chem.

[25] Sasaki Y, Araki T, Milbrandt J (2006). Stimulation of nicotinamide adenine dinucleotide biosynthetic pathways delays axonal degeneration after axotomy.. J Neurosci.

[26] Yan T, Feng Y, Zheng J, Ge X, Zhang Y (2009). Nmnat2 delays axon degeneration in superior cervical ganglia dependent on its NAD synthesis activity.. Neurochem Int Epub ahead of print.

[27] Ferri A, Sanes J. R, Coleman M. P, Cunningham J. M, Kato A. C (2003). Inhibiting axon degeneration and synapse loss attenuates apoptosis and disease progression in a mouse model of motoneuron disease.. Curr Biol.

[28] Wang M, Wu Y, Culver D. G, Glass J. D (2001). The gene for slow Wallerian degeneration (*Wld*
^S^) is also protective against vincristine neuropathy.. Neurobiol Dis.

[29] Martin D. P, Ito A, Horigome K, Lampe P. A, Johnson E. M (1992). Biochemical characterization of programmed cell death in NGF-deprived sympathetic neurons.. J Neurobiol.

[30] Kirkland R. A, Franklin J. L (2007). Rate of neurite outgrowth in sympathetic neurons is highly resistant to suppression of protein synthesis: role of protein degradation/synthesis coupling.. Neurosci Lett.

[31] Estridge M, Bunge R (1978). Compositional analysis of growing axons from rat sympathetic neurons.. J Cell Biol.

[32] Fainzilber M, Twiss J. L (2006). Tracking in the Wld^S^ - the hunting of the SIRT and the luring of the Draper.. Neuron.

[33] Eng H, Lund K, Campenot R. B (1999). Synthesis of beta-tubulin, actin, and other proteins in axons of sympathetic neurons in compartmented cultures.. J Neurosci.

[34] Berger F, Lau C, Dahlmann M, Ziegler M (2005). Subcellular compartmentation and differential catalytic properties of the three human nicotinamide mononucleotide adenylyltransferase isoforms.. J Biol Chem.

[35] Deckwerth T. L, Johnson E. M, (1994). Neurites can remain viable after destruction of the neuronal soma by programmed cell death (apoptosis).. Dev Biol.

[36] Burne J. F, Staple J. K, Raff M. C (1996). Glial cells are increased proportionally in transgenic optic nerves with increased numbers of axons.. J Neurosci.

[37] Finn J. T, Weil M, Archer F, Siman R, Srinivasan A (2000). Evidence that Wallerian degeneration and localized axon degeneration induced by local neurotrophin deprivation do not involve caspases.. J Neurosci.

[38] Whitmore A. V, Lindsten T, Raff M. C, Thompson C. B (2003). The proapoptotic proteins Bax and Bak are not involved in Wallerian degeneration.. Cell Death Differ.

[39] Laser H, Mack T. G, Wagner D, Coleman M. P (2003). Proteasome inhibition arrests neurite outgrowth and causes “dying-back” degeneration in primary culture.. J Neurosci Res.

[40] Lunn E. R, Perry V. H, Brown M. C, Rosen H, Gordon S (1989). Absence of Wallerian degeneration does not hinder regeneration in peripheral nerve.. Eur J Neurosci.

[41] Wang M. S, Davis A. A, Culver D. G, Glass J. D (2002). *Wld*
^S^ mice are resistant to paclitaxel (taxol) neuropathy.. Ann Neurol.

[42] Merianda T. T, Lin A. C, Lam J. S, Vuppalanchi D, Willis D. E (2008). A functional equivalent of endoplasmic reticulum and Golgi in axons for secretion of locally synthesized proteins.. Mol Cell Neurosci.

[43] Sasaki Y, Vohra B. P, Lund F. E, Milbrandt J (2009). Nicotinamide mononucleotide adenylyl transferase-mediated axonal protection requires enzymatic activity but not increased levels of neuronal nicotinamide adenine dinucleotide.. J Neurosci.

[44] Suzuki K, Koike T (2007). Resveratrol abolishes resistance to axonal degeneration in slow Wallerian degeneration (*Wld*
^S^) mice: activation of SIRT2, an NAD-dependent tubulin deacetylase.. Biochem Biophys Res Commun.

[45] Wang J, Zhai Q, Chen Y, Lin E, Gu W (2005). A local mechanism mediates NAD-dependent protection of axon degeneration.. J Cell Biol.

[46] Miller B. R, Press C, Daniels R. W, Sasaki Y, Milbrandt J (2009). A dual leucine kinase-dependent axon self-destruction program promotes Wallerian degeneration.. Nat Neurosci.

[47] Spencer M. J, Guyon J. R, Sorimachi H, Potts A, Richard I (2002). Stable expression of calpain 3 from a muscle transgene in vivo: immature muscle in transgenic mice suggests a role for calpain 3 in muscle maturation.. Proc Natl Acad Sci U S A.

[48] Zhai R. G, Cao Y, Hiesinger P. R, Zhou Y, Mehta S. Q (2006). *Drosophila* NMNAT maintains neural integrity independent of its NAD synthesis activity.. PLoS Biol.

[49] Zhai R. G, Zhang F, Hiesinger P. R, Cao Y, Haueter C. M (2008). NAD synthase NMNAT acts as a chaperone to protect against neurodegeneration.. Nature.

[50] De Vos K. J, Grierson A. J, Ackerley S, Miller C. C (2008). Role of axonal transport in neurodegenerative diseases.. Annu Rev Neurosci.

[51] Minoshima S, Cross D (2008). In vivo imaging of axonal transport using MRI: aging and Alzheimer's disease.. Eur J Nucl Med Mol Imaging.

[52] Wong M. L, Yen Y. R (1998). Protein synthesis in pseudorabies virus-infected cells: decreased expression of protein kinase PKR, and effects of 2-aminopurine and adenine.. Virus Res.

[53] Bendotti C, Calvaresi N, Chiveri L, Prelle A, Moggio M (2001). Early vacuolization and mitochondrial damage in motor neurons of FALS mice are not associated with apoptosis or with changes in cytochrome oxidase histochemical reactivity.. J Neurol Sci.

[54] Fischer L. R, Culver D. G, Davis A. A, Tennant P, Wang M (2005). The *Wld*
^S^ gene modestly prolongs survival in the SOD1G93A fALS mouse.. Neurobiol Dis.

[55] Campenot R. B (1992). Construction and use of compartmented cultures..

[56] Adalbert R, Gillingwater T. H, Haley J. E, Bridge K, Beirowski B (2005). A rat model of slow Wallerian degeneration (WldS) with improved preservation of neuromuscular synapses.. Eur J Neurosci.

