# Supplementary figures and images for: Endogenous Nmnat2 Is an Essential Survival Factor for Maintenance of Healthy Axons
Source: PLoS Biol. 2010 Jan 26;8(1):e1000300. doi: 10.1371/journal.pbio.1000300 (PMC2811159; doi:10.1371/journal.pbio.1000300)

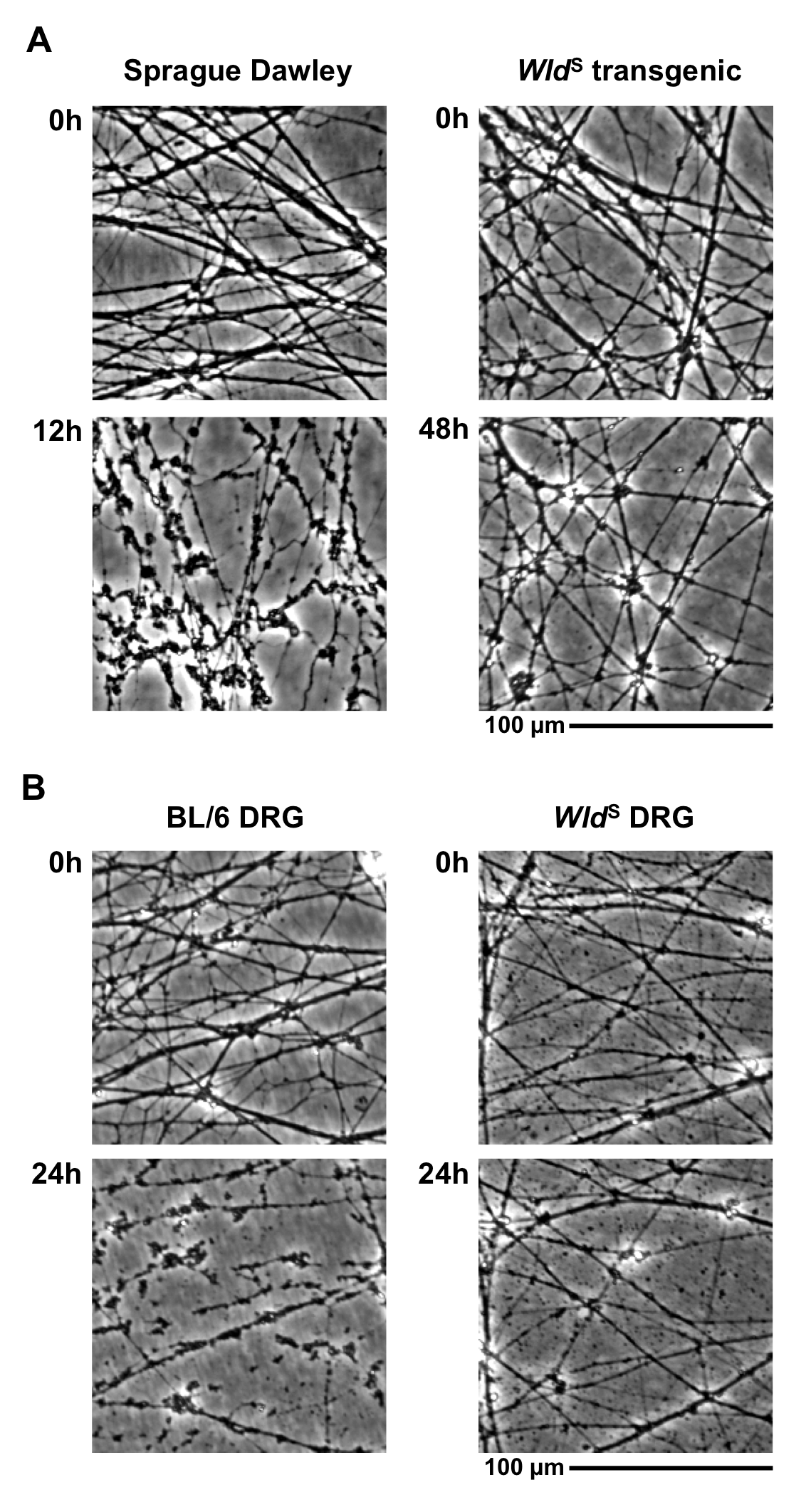

Supplement: Figure S1 — Suppression of protein synthesis induces Wallerian-like neurite degeneration in rat SCG and mouse DRG explant cultures. Representative bright-field images of distal neurites in (A) wild-type (Sprague Dawley) or Wld S transgenic rat SCG explant cultures and (B) wild-type or Wld S mouse DRG explant cultures each treated with 10 µM emetine. Images were captured at the indicated times after emetine addition and are representative of multiple fields in three independent experiments. DRG neurites appear to be more resistant than SCG neurites to the effects of translation inhibitors as 20 µg/ml CHX was required to consistently induce neurite degeneration by 24 h (unpublished data). This is consistent with DRG neurites also undergoing Wallerian degeneration at a slower rate than SCG neurites (compare Figure S2B with [25]). (1.78 MB TIF) [file pbio.1000300.s001.tif]

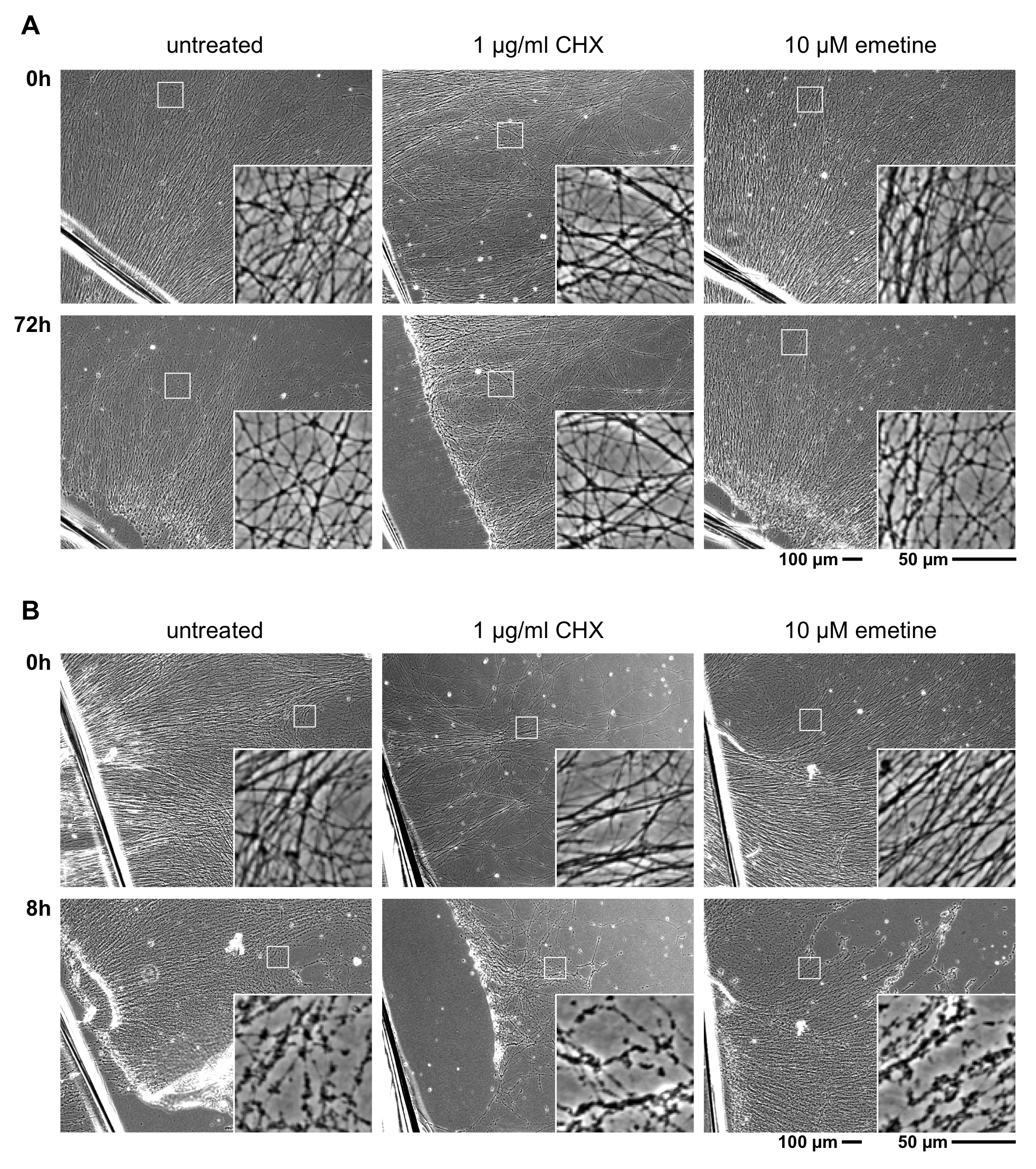

Supplement: Figure S2 — Neither Wld S-mediated protection of transected SCG neurites nor Wallerian degeneration itself requires localized translation in neurites. Representative bright-field images of transected neurites from (A) Wld S or (B) wild-type (BL/6) mouse SCG explant cultures treated with vehicle (untreated), 1 µg/ml CHX, or 10 µM emetine. Inhibitors were added just before transection and images captured at the times indicated after cut. Part of the cut site is visible in the bottom left-hand corner. Increased magnification of framed regions is shown for better visualization of neurites. Healthy-looking Wld S neurites occasionally detached from the culture dish prior to 72 h after cut probably due to handling of the cultures. Images are representative of multiple fields in two or more independent experiments. (4.79 MB TIF) [file pbio.1000300.s002.tif]

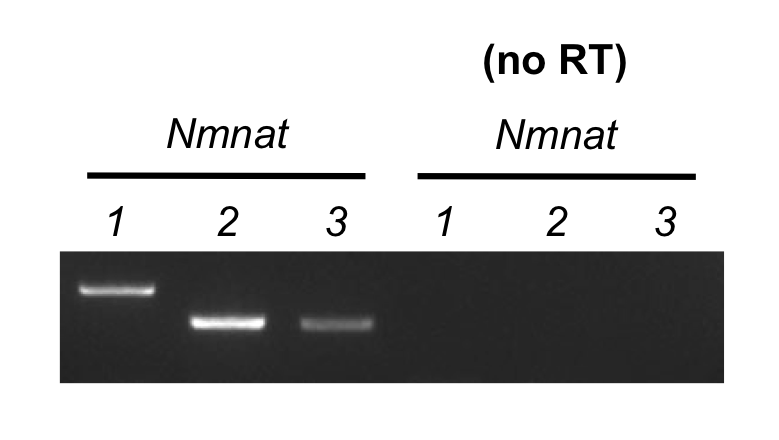

Supplement: Figure S3 — RT-PCR analysis indicates that mRNAs of all three Nmnat isoforms are expressed in SCG neurons. Amplification products were resolved on a 2% ethidium bromide-stained agarose gel. The image is representative of three independent experiments. Reactions with no reverse transcriptase (no RT) were included to confirm no DNA contamination. The amplification kinetics of each set of primers are approximately equivalent (unpublished data), suggesting that Nmnat2 mRNA may be expressed at slightly higher levels than Nmnat1 and Nmnat3 mRNAs. (0.07 MB TIF) [file pbio.1000300.s003.tif]

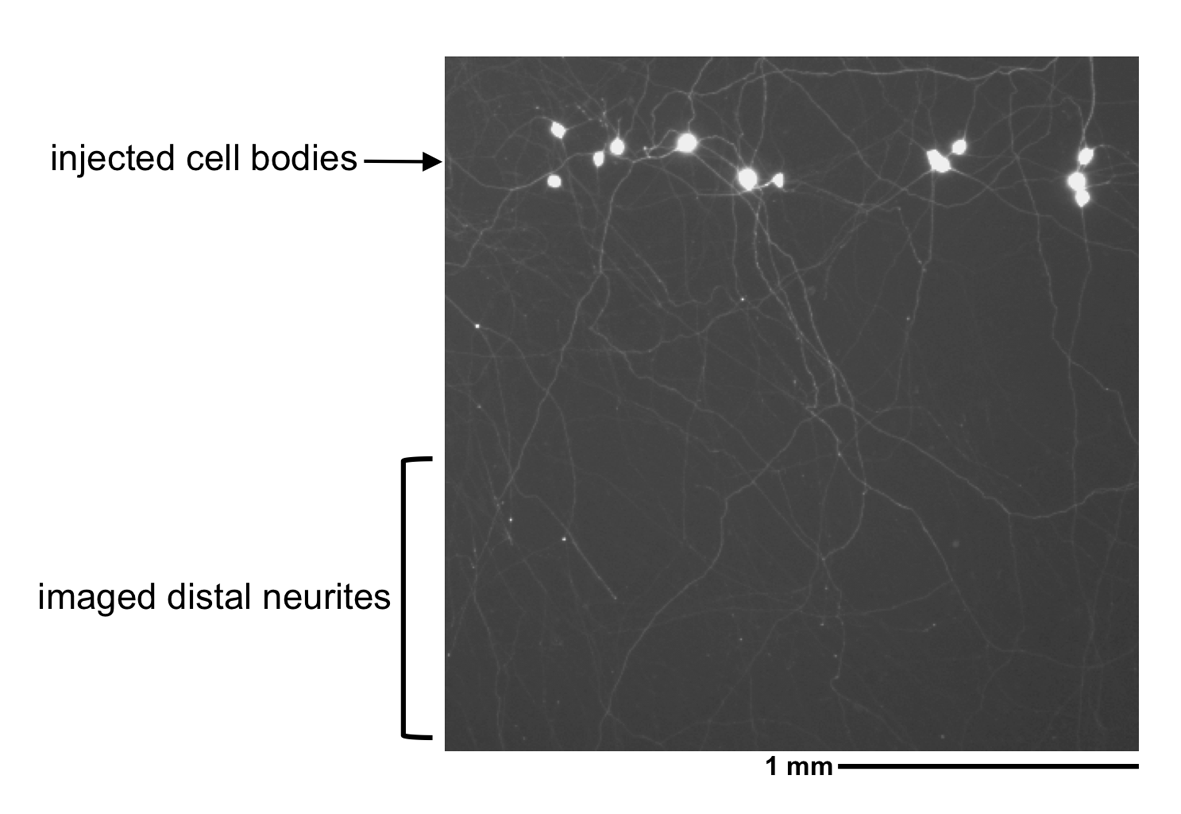

Supplement: Figure S4 — Microinjection-based strategy for assessing the effects of siRNAs in SCG neurons. Nuclei were injected with a mix containing siRNA (here injected with siControl) and pDsRed2-N1 (50 ng/µl). High expression of DsRed2 allowed clear visualization of injected neurons and complete neurite projections 24 h after injection. The intensity of DsRed2-labeling increases over subsequent days. A line of neuronal cell bodies was injected to simplify differentiation of the proximal and distal portions of neurites. Distal neurites were imaged to allow their health to be monitored 24, 48, and 72 h after injection. (0.37 MB TIF) [file pbio.1000300.s004.tif]

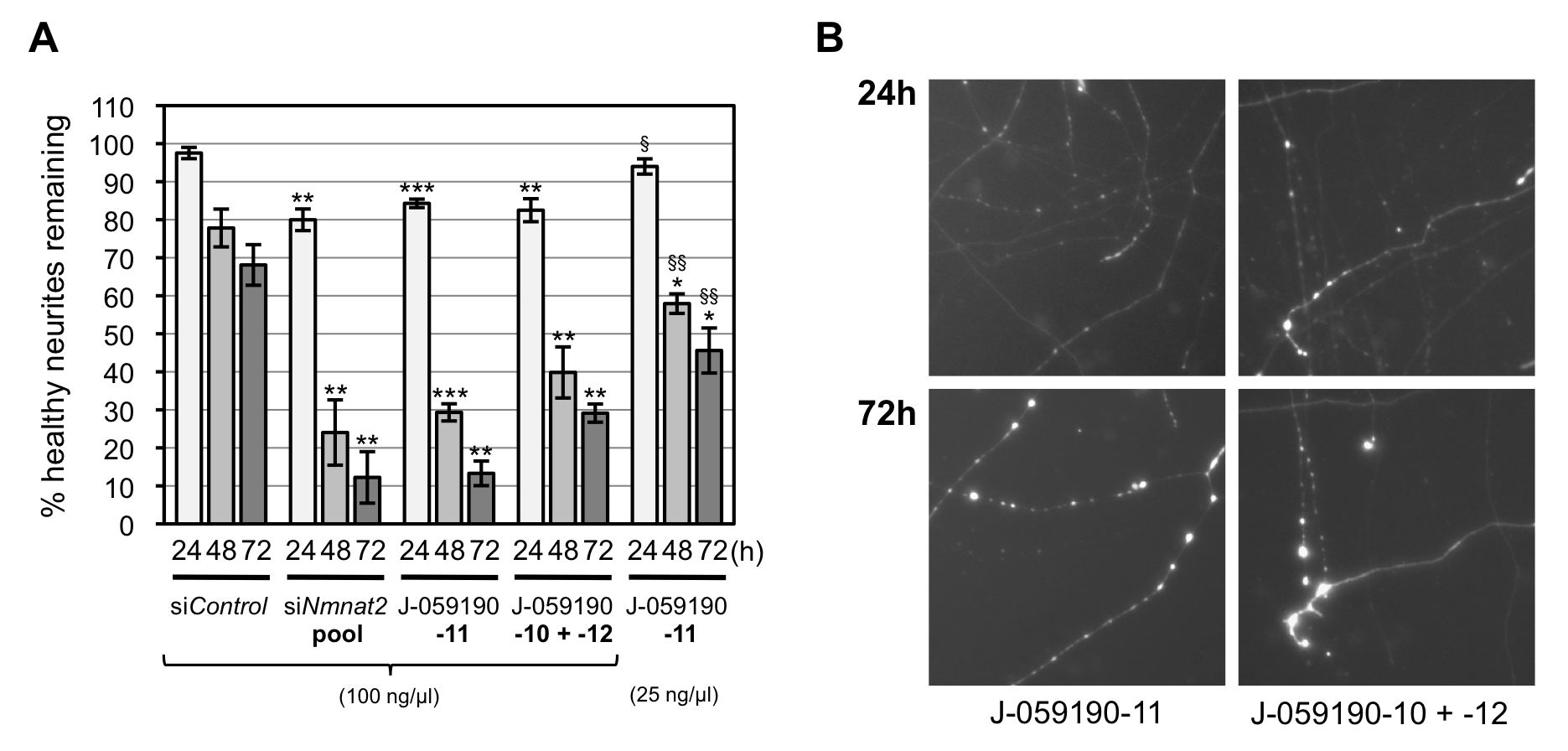

Supplement: Figure S5 — Neurite degeneration triggered by Nmnat2 siRNA is a result of Nmnat2 knock-down rather than an off-target effect. (A) Effects of the four siRNAs making up the siNmnat2 pool – J-059190-09, -10, -11, and -12—injected individually or in subpools (together with pDsRed2-N1 at 50 ng/µl). Neurite survival is shown as a percentage of the total number of DsRed2-labeled neurites (healthy and abnormal) at 24 h and was quantified from three independent experiments combining data from multiple fields (error bars = ±S.E.M.). J-059190-11 alone (100 ng/µl) and J-059190-10 and J-059190-12 in combination (50 ng/µl each) caused significant loss of distal neurites comparable to the siNmnat2 pool (**p<0.01, ***p<0.001, t test versus equivalent time point for siControl). J-059190-09, -10, and -12 caused only limited neurite degeneration individually (unpublished data). J-059190-09 injected at 25 ng/µl, equivalent to its contribution in the siNmnat2 pool (at 100 ng/µl), also caused significant neurite loss (*p<0.05, t test versus equivalent time point for siControl), but this was significantly reduced compared to injection at 100 ng/µl (§ p<0.05, §§ p<0.01, t test J-059190-11 at 25 ng/µl versus 100 ng/µl at equivalent time points). (B) Representative fluorescent images of DsRed2-labeled neurites of wild-type (BL/6) SCG neurons 24 and 72 h after injection with J-059190-11 or J-059190-10+-12 (together with pDsRed2-N1). Abnormal neurite morphology and neurite loss at 72 h is identical to that seen following injection of the siNmnat2 pool (Figure 4). (0.56 MB TIF) [file pbio.1000300.s005.tif]

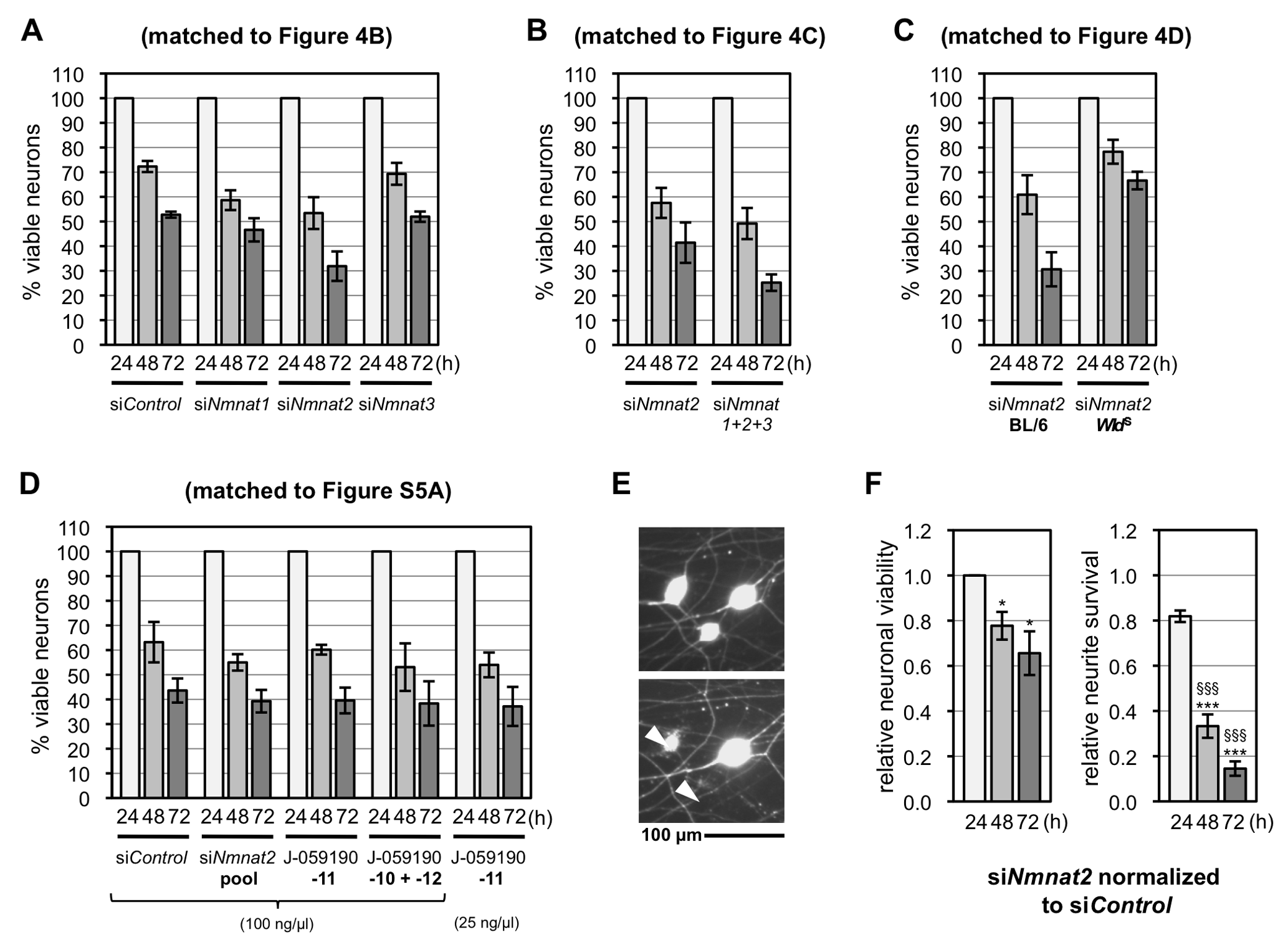

Supplement: Figure S6 — Loss of neuron viability after injection with siRNA and the DsRed2 expression vector at high concentration. (A–D) Quantification of neuron viability matched to the analyses of neurite survival in Figure 4 and Figure S5 as indicated. The number of neurons with normal gross morphology remaining at each time point is shown as a percentage of those present at 24 h (error bars = ±S.E.M.). Injection of siNmnat2 appeared to cause a decrease in neuronal viability relative to siControl in some experiments (A–C) and this was prevented in Wld S neurons (C). Individual siRNAs from the siNmnat2 pool had equivalent effects on neuronal viability (D). (E) Neuron viability was scored based on gross morphology (in bright-field and DsRed2 imaging) of the injected neuron cell bodies. Two of the three injected neurons shown with normal morphology in the top panel appear abnormal 24 h later (arrowheads, bottom panel). Assessment based on gross morphology closely matches other indicators of cell viability (see Figure 5B). (F) Comparison between the amounts of neuron and neurite loss induced by siNmnat2. Percentages of viable neurons and healthy neurites at each time point after siNmnat2 injection were normalized to matched siControl percentages in all paired experiments performed (n = 8) and are expressed as a fraction of siControl values (error bars = ±S.E.M). The significant reduction in neurite health following injection of siNmnat2 is proportionately far greater than the small but significant reduction in neuronal viability (*p<0.05, ***p<0.001, t test siNmnat2 versus siControl at equivalent time points; §§§ p<0.001, t test siNmnat2 neurite survival versus siNmnat2 neuron survival at equivalent time points). In contrast, neuron loss exceeds neurite degeneration with all other siRNA pools. This is presumably due to preferential loss of neurons that do not project neurites into areas where degeneration is assessed. This may be because neurons with shorter neurites are more susceptible t [file pbio.1000300.s006.tif]

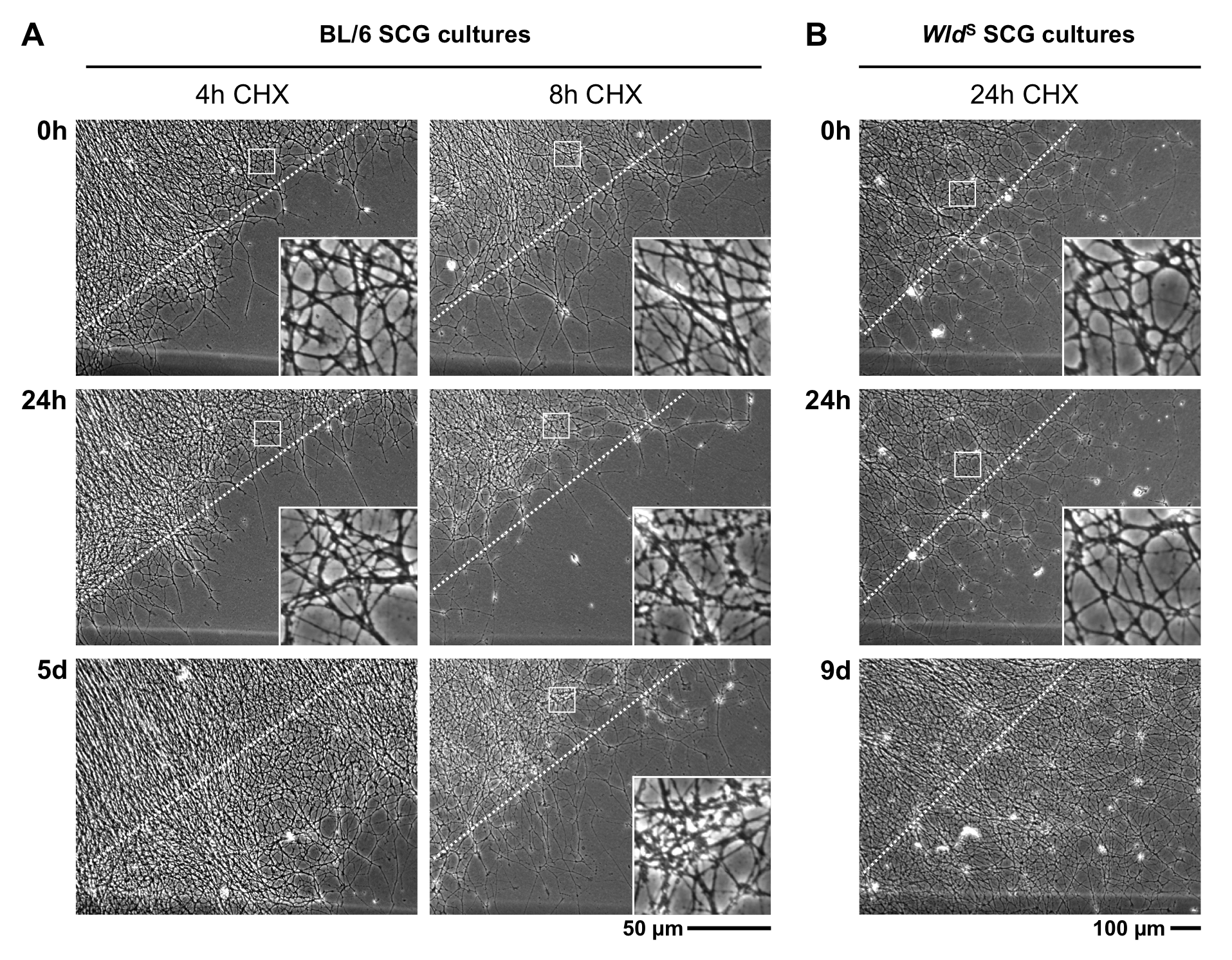

Supplement: Figure S7 — Wild-type SCG neurites become committed to degenerate after approximately 6 h of protein synthesis suppression and this is delayed by WldS. Representative bright-field images of distal neurites from (A) wild-type (BL/6) or (B) Wld S mouse SCG explant cultures treated with 1 µg/ml CHX for 4, 8, or 24 h before inhibitor removal. Images of the same field of neurites were captured at the indicated times after initial addition of the inhibitor (0 h). Framed regions are magnified for better visualization of neurite morphology. A dashed white line provides a reference point against which relative neurite extension can be assessed. Images for the 4 h and 8 h treatments of BL/6 cultures are representative of nine fields in three independent experiments, and images for the 24 h treatment of Wld S cultures are representative of 8 out of 10 fields in four independent experiments. An intermediate 6 h treatment of BL/6 neurites resulted in a mixed outcome; neurites became significantly blebbed by 24 h in four out of nine fields in three independent experiments. A 48 h treatment of Wld S cultures blocked neurite outgrowth in all six fields in three independent experiments and caused blebbing by day 9 in four out of six fields (unpublished data). (2.96 MB TIF) [file pbio.1000300.s007.tif]

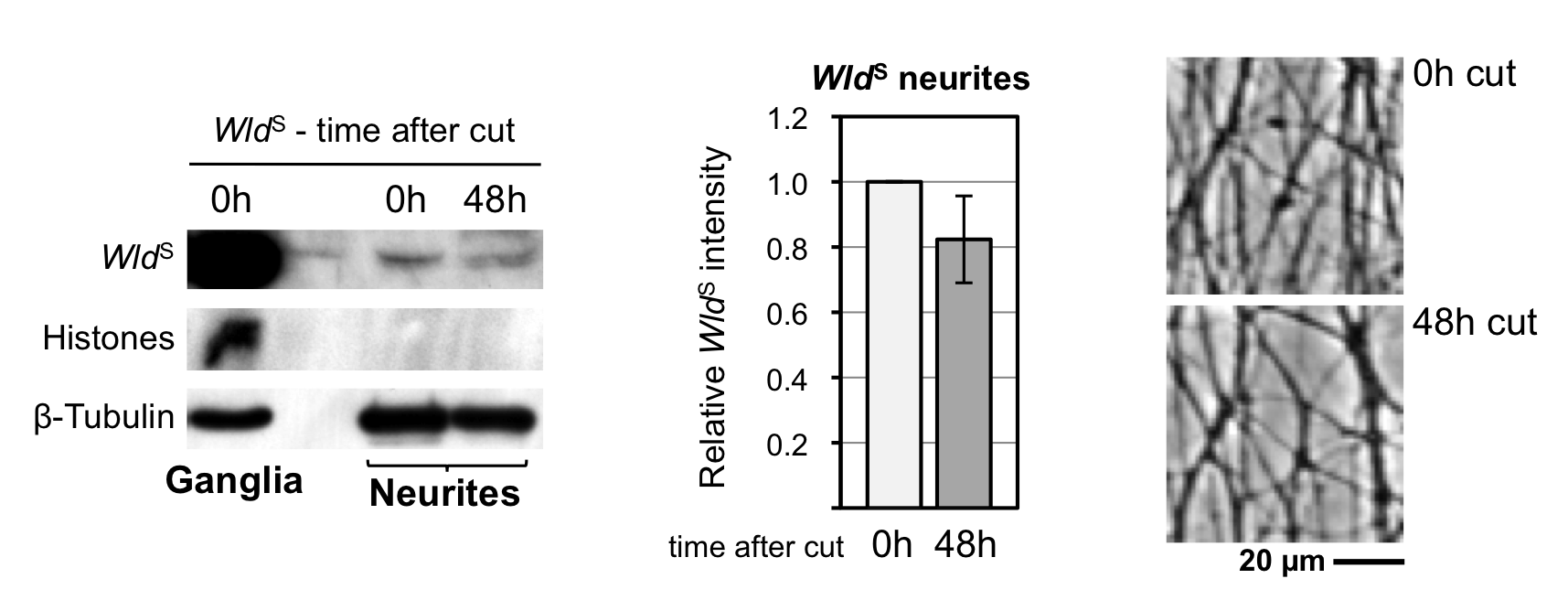

Supplement: Figure S8 — WldS levels are only slightly reduced in Wld S neurites 48 h after transection. Representative immunoblot showing levels of WldS in transected Wld S neurites just after cut (0 h) and 48 h later. ß-Tubulin acts as a loading control. Absence of the 16 kDa core Histones band in neurite extracts confirms there is no detectable contamination with SCG cell bodies or non-neuronal cells. Each lane represents material collected from SCG explant cultures derived from 8–10 ganglia (below the threshold for consistent detection of Nmnat2). WldS band intensity at 48 h is plotted (centre) as a fraction of that at 0 h after normalization to ß-Tubulin and was quantified from three independent experiments (error bars = ±S.E.M.). Images of the same field of neurites (right) show representative transected neurite morphology at 0 h and 48 h. (0.36 MB TIF) [file pbio.1000300.s008.tif]

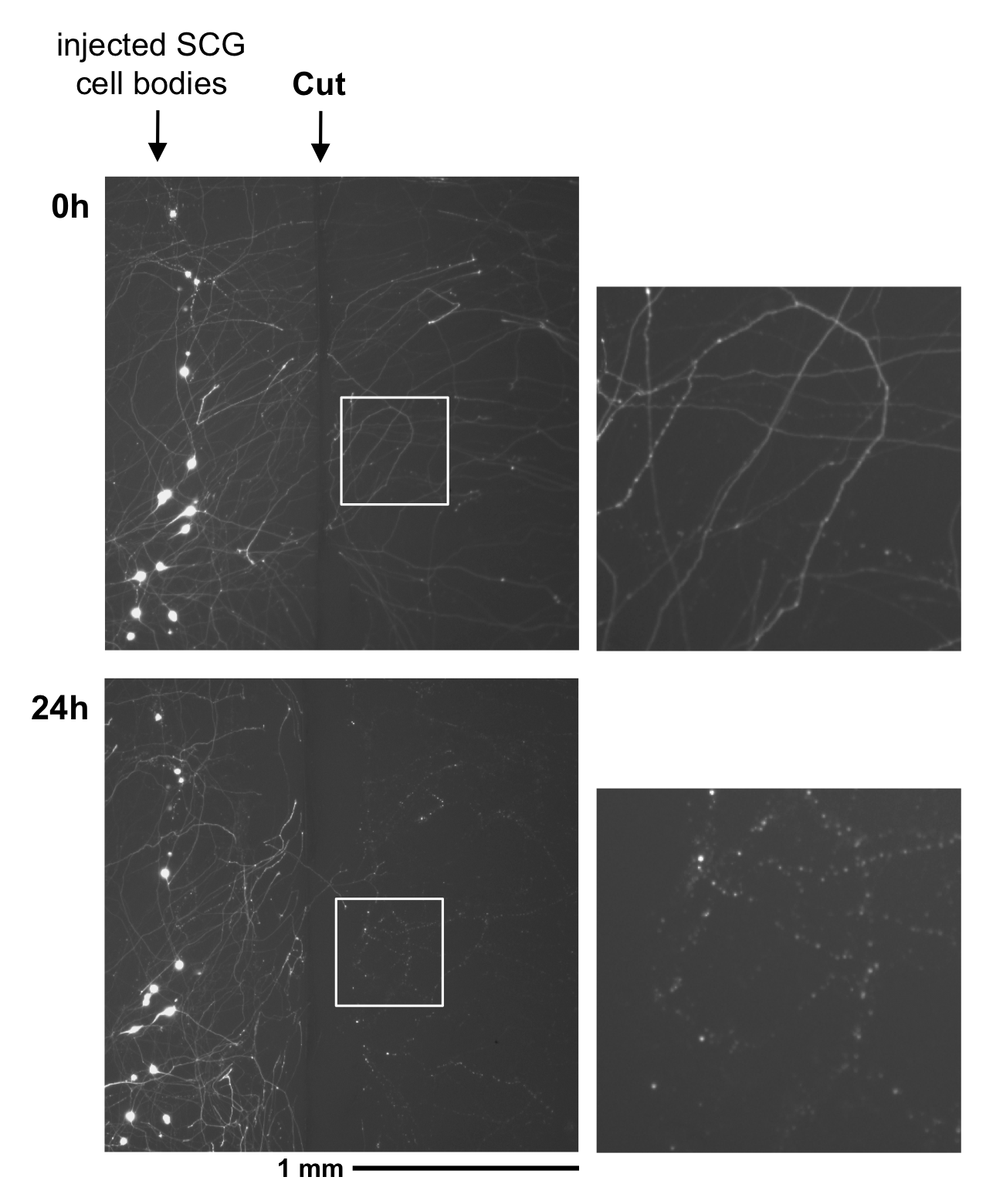

Supplement: Figure S9 — Assessing injury-induced degeneration of DsRed2-labeled SCG neurites. Representative fluorescent images of the same field of transected DsRed2-labeled neurites of wild-type (BL/6) neurons injected with 1 ng/µl empty FLAG vector (FLAG-empty) and pDsRed2-N1 (50 ng/µl) immediately after cut (0 h) and 24 h later. Lower magnification images show a line of injected neuronal cell bodies and the neurite network projecting from them. This pattern of injection facilitated transection such that all injected neurons and their proximal neurites were located on the opposite side of the cut to the transected distal neurites. The location of the cut site is indicated. All neurites disconnected from their cell bodies have degenerated by 24 h. Identical results were obtained with 50 ng/µl FLAG-empty. Increased magnification of the framed regions is shown for better visualization of neurites. (0.85 MB TIF) [file pbio.1000300.s009.tif]
